# Synthesis and Studies of the Inhibitory Effect of Hydroxylated Phenylpropanoids and Biphenols Derivatives on Tyrosinase and Laccase Enzymes

**DOI:** 10.3390/molecules25112709

**Published:** 2020-06-11

**Authors:** Maria Antonietta Dettori, Davide Fabbri, Alessandro Dessì, Roberto Dallocchio, Paola Carta, Claudia Honisch, Paolo Ruzza, Donatella Farina, Rossana Migheli, Pier Andrea Serra, Roberto A. Pantaleoni, Xenia Fois, Gaia Rocchitta, Giovanna Delogu

**Affiliations:** 1 Istituto di Chimica Biomolecolare, Consiglio Nazionale Ricerche, 07100 Sassari, Italy; mariaantonietta.dettori@cnr.it (M.A.D.); davidegaetano.fabbri@cnr.it (D.F.); alessandro.dessi@cnr.it (A.D.); robertonico.dallocchio@cnr.it (R.D.); paola.carta@cnr.it (P.C.); 2 Dipartimento di Scienze Chimiche, Università degli Studi di Padova, 35131 Padova, Italy; claudia.honisch@phd.unipd.it (C.H.); or paolo.ruzza@cnr.it (P.R.); 3 Istituto di Chimica Biomolecolare, Consiglio Nazionale Ricerche, 35131 Padova, Italy; 4 Dipartimento di Scienze Mediche, Chirurgiche e Sperimentali, Università degli Studi, 07100 Sassari, Italy; donatellafarina@tiscali.it (D.F.); rmigheli@uniss.it (R.M.); paserra@uniss.it (P.A.S.); 5 Istituto di Ricerca sugli Ecosistemi Terrestri, Consiglio Nazionale Ricerca, 07100 Sassari, Italy; roberto.pantaleoni@iret.cnr.it (R.A.P.); xeniafois@hotmail.it (X.F.); 6 Dipartimento di Agraria, Università degli Studi, 07100 Sassari, Italy

**Keywords:** oxidase and polyphenol oxidase, polyphenols, tyrosinase inhibitors, chitin inhibitors, SAR studies, biosensors, spectrophotometric assay, viability

## Abstract

The impaired activity of tyrosinase and laccase can provoke serious concerns in the life cycles of mammals, insects and microorganisms. Investigation of inhibitors of these two enzymes may lead to the discovery of whitening agents, medicinal products, anti-browning substances and compounds for controlling harmful insects and bacteria. A small collection of novel reversible tyrosinase and laccase inhibitors with a phenylpropanoid and hydroxylated biphenyl core was prepared using naturally occurring compounds and their activity was measured by spectrophotometric and electrochemical assays. Biosensors based on tyrosinase and laccase enzymes were constructed and used to detect the type of protein-ligand interaction and half maximal inhibitory concentration (IC_50_). Most of the inhibitors showed an IC_50_ in a range of 20–423 nM for tyrosinase and 23–2619 nM for laccase. Due to the safety concerns of conventional tyrosinase and laccase inhibitors, the viability of the new compounds was assayed on PC12 cells, four of which showed a viability of roughly 80% at 40 µM. In silico studies on the crystal structure of laccase enzyme identified a hydroxylated biphenyl bearing a prenylated chain as the lead structure, which activated strong and effective interactions at the active site of the enzyme. These data were confirmed by in vivo experiments performed on the insect model *Tenebrio molitur*.

## 1. Introduction

**Figure 1:**
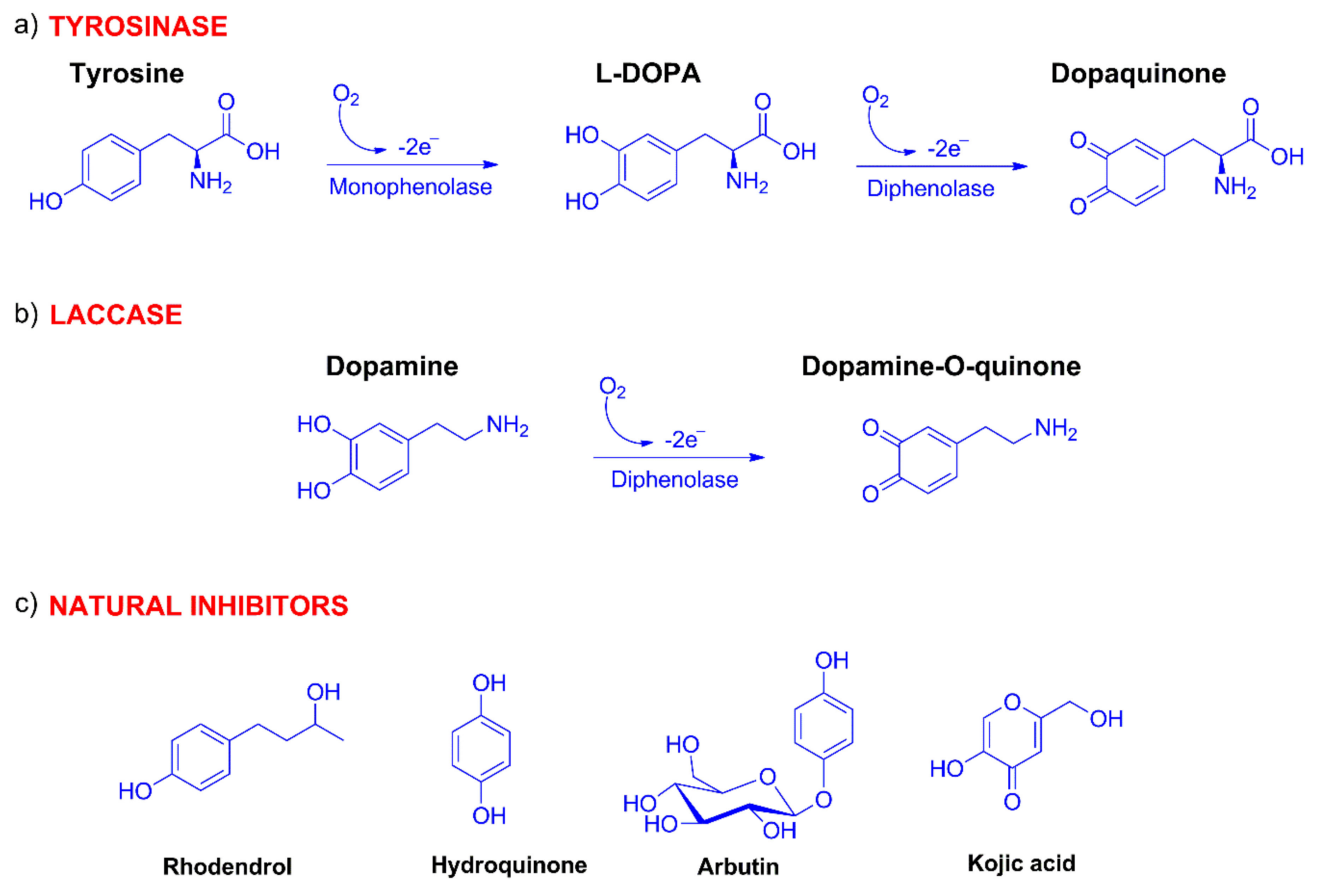
Enzyme reactions of (**a**) tyrosinase and (**b**) laccase and (**c**) natural inhibitors.

Tyrosinase—also referred to as monophenol monooxygenases (E.C. 1.14.18.1)—is a key enzyme in melanin biosynthesis and is involved in determining the color of mammalian skin and hair, the enzymatic browning of plant-derived food, and the molting process of insects [1,2]. Tyrosinase contains a binuclear, active copper site responsible for catalyzing two distinct reactions involving molecular oxygen. These reactions are the hydroxylation of monophenols to *o*-diphenols (monophenolase activity) and the oxidation of *o*-phenols to *o*-quinones (diphenolase activity), as described in Figure 1.

In the central domain of tyrosinase, in all living organisms originating from a common ancestral gene, the two copper ions activate interactions with oxygen atoms [3]. Each copper ion is bound to three conserved histidine residues which are essential for the stabilization of the active site. The natural substrate of tyrosinase, the amino acid L-tyrosine, is also the melanin precursor. An abnormal lack of melanin causes albinism, whereas an excessive accumulation of melanin can result in hyperpigmentation, melanoma in humans, as well as undesirable browning in fruits and vegetables [4,5,6]. Tyrosinase oxidizes dopamine, which forms melanin in the brain, and thus plays a role in the first stage of Parkinson’s and other neurodegenerative diseases [7]. Moreover, the enzyme product melanin has been shown to compromise the activity of traditional antibiotics [8].

Laccase, a phenoloxidase (PO, EC 1.10.3.2), is a member of multicopper oxidases family that has been found in higher plants, fungi, bacteria and insects [9,10]. It catalyzes the monoelectronic oxidation of substrates at the expense of molecular oxygen (Figure 1) [11]. Laccases have a wide substrate range, and thus are used for industrial purposes and/or bioremediation processes [12].

Like tyrosinase, laccase was found in the cuticle of many insect species, and plays a primary role in insect development and immunity, such as cuticle sclerotization, wound healing and defense against foreign pathogens, and thus provides a new target for insect pest control [13,14].

Several crystal structures of different tyrosinases and laccases from various sources have been isolated and studied [15,16,17,18], however a high-resolution structure of human tyrosinase has not been fully characterized due to its flexible structural feature [19].

Laccases and tyrosinases are two groups of phenoloxidases that catalyze the transformation of a large number of phenolic and non-phenolic aromatic compounds. Investigating the inhibitors of both enzymes would thus lead to the development of a wide range of compounds ranging from novel skin whitening agents, to medicinal products, to anti-browning substances and to compounds capable of controlling harmful insects and bacteria [20,21,22]. From a mechanistic point of view, a commercially suitable tyrosinase/laccase inhibitor would bind reversibly to the enzyme and reduce its catalytic capacity [23]. Although many tyrosinase and laccase inhibitors have been identified and their activity has been measured even at low concentrations, few have been put into practical use. The main reasons are the lack of the specific activity, instability and safety concerns [24,25].

Commercial alternatives to hydroquinone and rhodendrol, two tyrosinase inhibitors both banned from the market due to health concerns [26], include natural-occurring compounds such as kojic acid [5-hydroxy-2-(hydroxymethyl)-4*H*-pyran-4-one] and arbutin (hydroquinone-*O*-β-d-glucopyranoside) (Figure 1) [27,28]. Unfortunately, neither compound offers a satisfactory safe and effective replacement [29]. 

**Figure 2:**
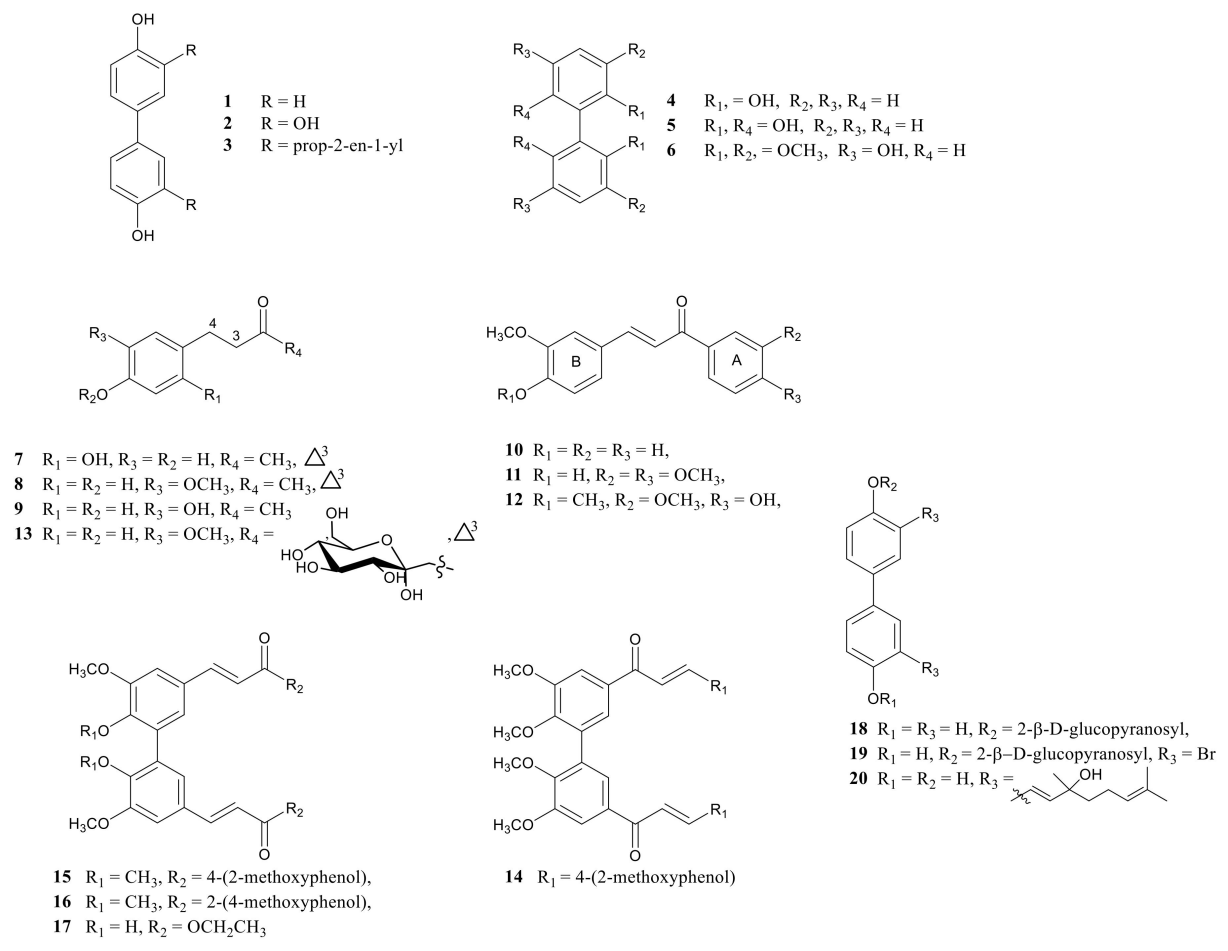
Hydroxylated phenylpropanoids and biphenols derivatives.

However, compound **1** (4,4′-dihydroxy-biphenyl), a biphenol produced by cyanobacteria and known as a radical scavenger, has been found to be a potent tyrosinase inhibitor—and more effective than kojic acid and arbutin (Figure 1 and Figure 2) [30]. It interacts with tyrosinase enzyme as a classical reversible competitive inhibitor.

Structurally, compound **1** belongs to the class of hydroxylated biphenyls widely distributed in the plant kingdom [31]. Hydroxylated biphenyls play an important role in biosystems due to their unique pharmacophore structure that is made up of two aromatic rings bridged by a single C–C bond [32]. The high level of specificity of the hydroxylated biphenyls scaffold with the catalytic domain of different proteins suggests that the structure could be an effective template for the discovery and design of new protein targets [33,34,35].

In our continuing research on the synthesis of hydroxylated biphenyls with interesting stereochemical and biologic features [36,37,38,39,40], we recently prepared a set of C_2_-symmetry biphenols (i.e., compounds **2**–**6**) structurally related to compound **1** and evaluated their tyrosinase inhibition by spectrophotometric and electrochemical assays, using a tyrosinase biosensor for the electrochemical assays [41]. Some of the molecules reported here (compounds **2**, **3**, **5** and **6**) showed a higher tyrosinase inhibitory activity than that of biphenol **1** (Figure 2).

The molecular basis of the new inhibitors mainly relies on an α,β-unsaturated carbonyl function conjugated to an aromatic scaffold which confers complete electron delocalization to the molecule thus reducing its redox potential [42,43,44]. This geometric motif is highly susceptible to electron transfer reactions with the amino acid residues of the catalytic domain of tyrosinase and laccase proteins [45,46].

With this in mind—and inspired by recent articles on new tyrosinase and laccase phenolic inhibitors—the aim of the present work is to prepare a small library of compounds with unsaturated chains whose structures are reminiscent of hydroxylated phenyl propanoids and biphenols. The effect of the compounds on the viability of PC12 cells was also assayed. A set of biosensors based on tyrosinase and laccase enzymes was constructed, and the inhibitory activity of the compounds was detected separately. Biosensor detection enabled us to investigate the reversibility of the inhibitors, the type of protein-ligand interactions that affect the catalytic domain, and lastly the IC_50_ of the new compounds. A computational study on the interactions between a laccase crystal data set and a potential cuticle inhibitor was performed in accordance with in vivo assays carried out on mealworm larvae of the insect model *Tenebrio molitor*.

## 2. Results

### 2.1. Synthesis of Compounds

Figure 2 shows a set of phenols and hydroxylated biphenyls, compounds **7, 8** and **10**–**17**, bearing an α,β-unsaturated carbonyl function, which were prepared under straightforward and sustainable procedures. Three biphenyl-analogs of **1**, compounds **18**–**20**, embedded with prenylated or glycosylated units were also prepared.

**Scheme 1:**
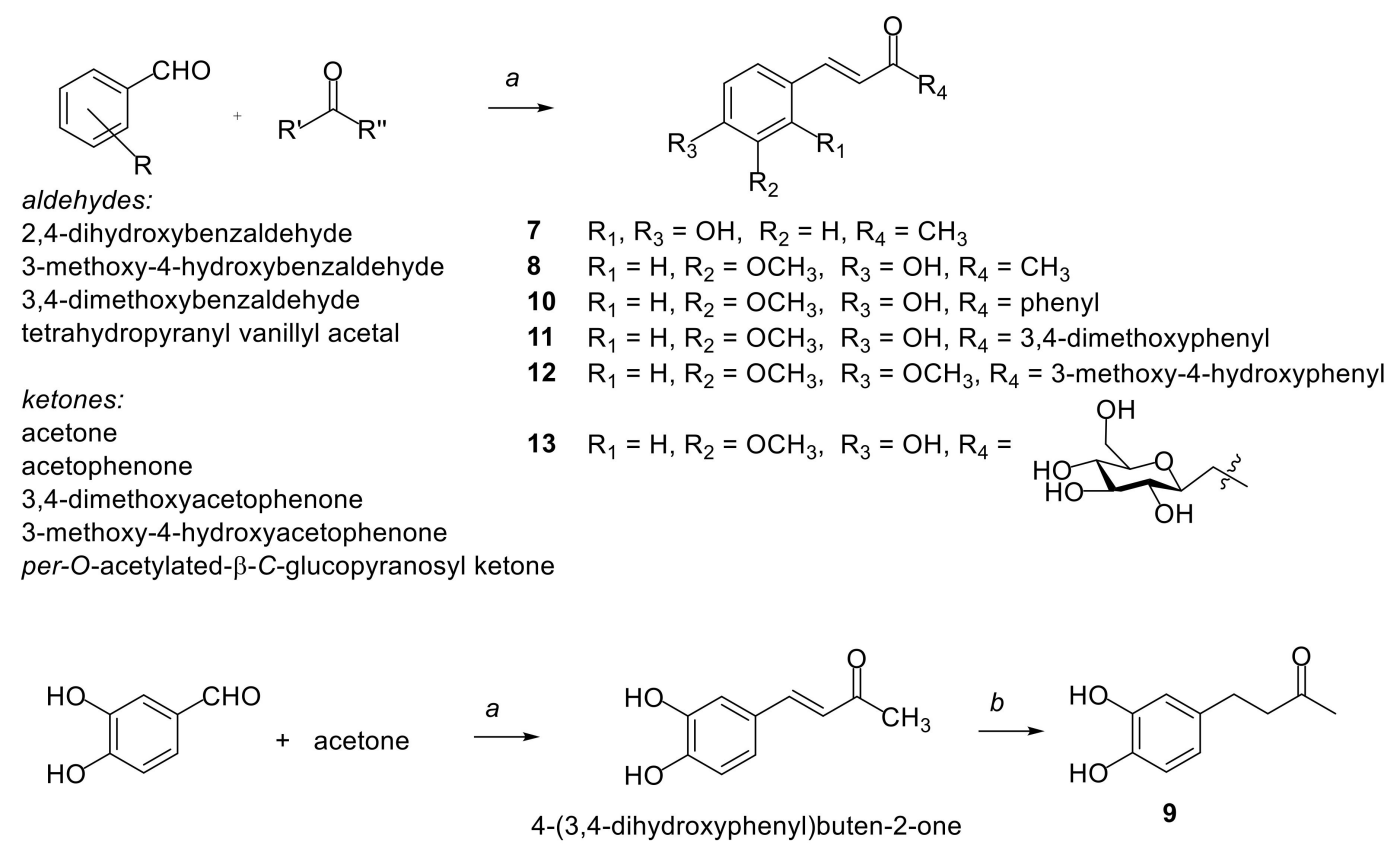
Reagents and conditions: (**a**) for compound **7**: NaOH**,** acetone, 12 h at 50 °C**;** for compounds **8** and **9**: NaOH**,** acetone, 12 h at rt; for compound **10**: LiOH, MeOH, 6 days at rt; for compound **11**: KOH, MeOH, 12 h at rt; for compound **12**: KOH/H_2_O, MeOH, 96 h at rt; for compound **13**: pyrrolidine, CH_2_Cl_2_, 72 h then PPTS, 3 h at rt then MeONa/MeOH, 10 min at rt; (**b**) Pd/C 10%, EtOAc, 3 h at rt.

Starting with an appropriate ketone and aldehyde the Claisen-Schmidt condensation reaction was applied to synthetise compounds **7**–**16**. This involves the reaction of a ketone with an aldehyde under basic conditions with the simultaneous loss of a water molecule. Compound **7** was prepared with a more straightforward and sustainable procedure than that the one used in the literature for the synthesis of this compound [43] producing a 60% yield, whereas compounds **8**–**11** were prepared as described in the literature (Scheme 1) [40,47,48,49].

**Scheme 2:**
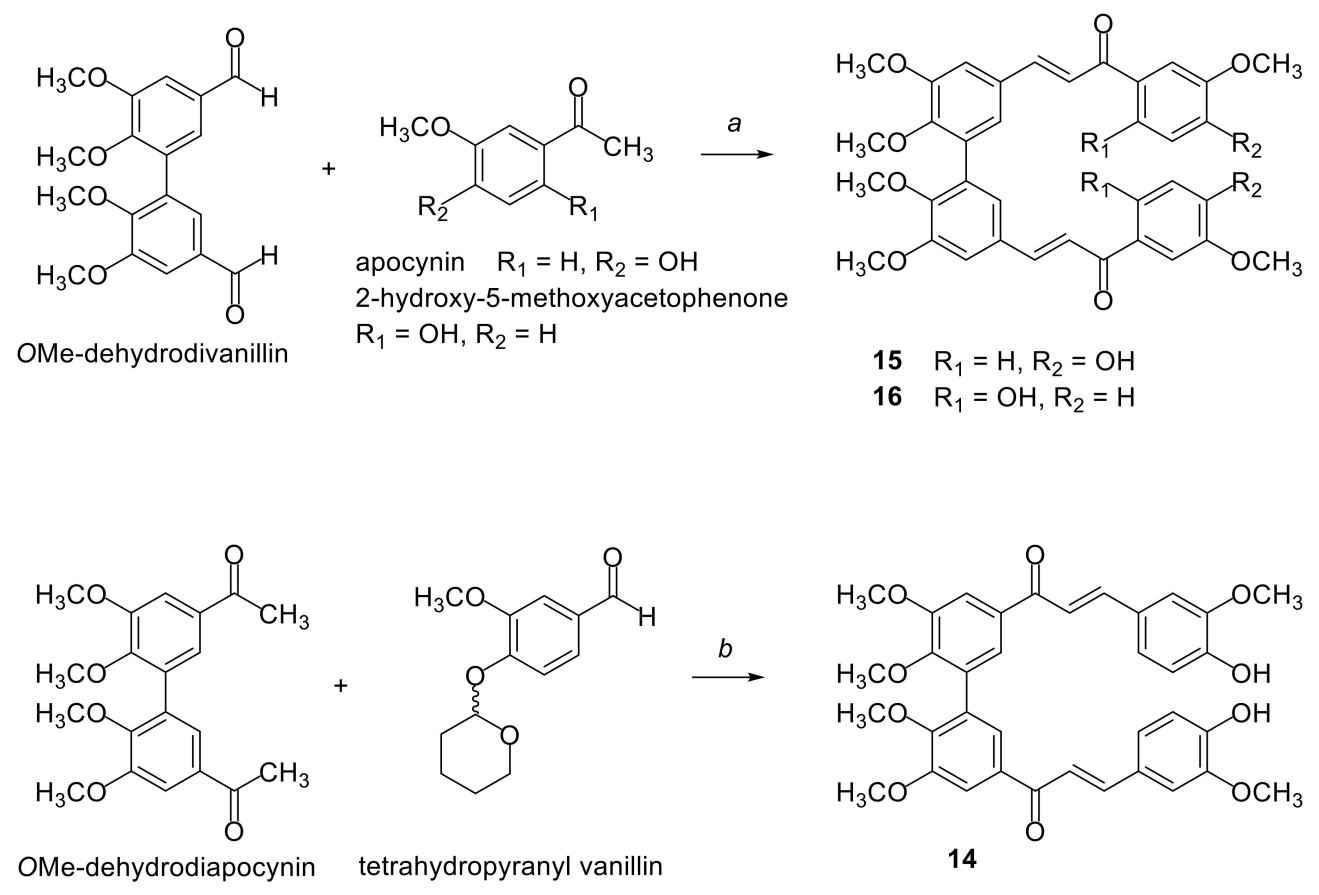
Reagents and conditions: (**a**) KOH/H_2_O, MeOH, 3 h at 0 °C, then 96 h at rt; (**b**) KOH/H_2_O, MeOH, 3 h at 0 °C → 12 h at rt, then *p*-TsOH, 2 h at rt.

Dimer of chalcone **11**, biphenyl **14**, was prepared starting with the reaction of *O*Me-dehydrodiapocynin and four equivalents of tetrahydropyranyl vanillyl acetal (Scheme 2).

**Scheme 3:**
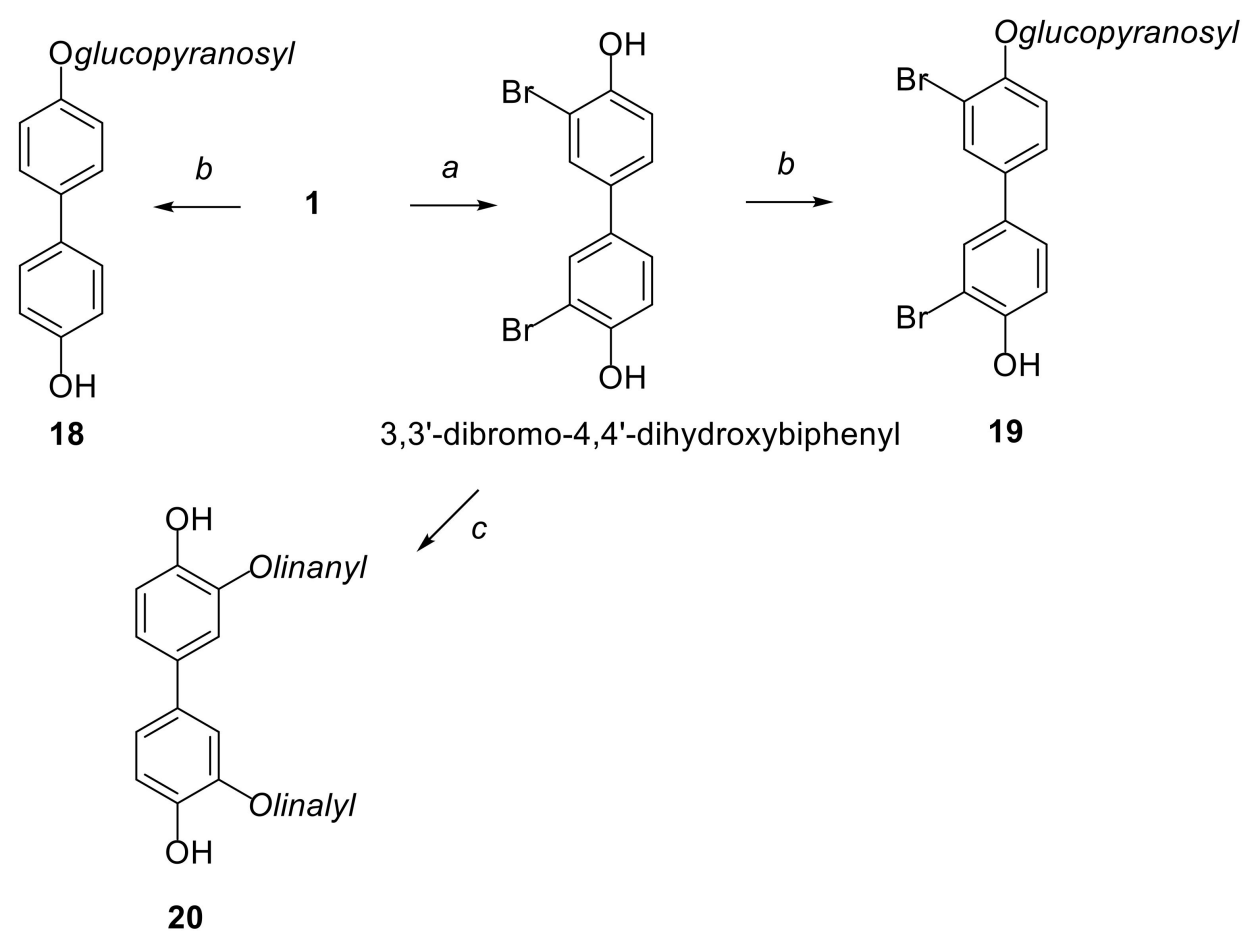
Reaction and conditions: (**a**) Br_2_, CH_2_Cl_2_, AcOH, 48 h at rt; (**b**) 2,3,4,6-tetra-*O*-acetyl-α-d-glucopyranosyl bromide and K_2_CO_3_ in acetone for 48 h at 50 °C, then MeOH: EtOAc 1:1 solution, MeONa at rt for 1 h; (**c**) DMF, Cs_2_CO_3_, [Pd(OAc)_2_] _3_, linalool, MW 10 min at 100 °C.

Both A rings of chalcones **12** and **15** have the core of apocynin (3-methoxy-4-hydroxyacetophenone) which in the presence of 3,4-dimethoxybenzaldehyde for chalcone **12** and *O*Me-dehydrodivanillin for biphenyl **15**, led to yields for the two chalcones of 85% and 70%, respectively (Scheme 1 and Scheme 2). Following the same procedure as applied for **15**, chalcone **16** was prepared with a 55% yield using 2-hydroxy-5-methoxyacetophenone as the starting ketone. The glycosylated compound **13** was obtained by the condensation reaction of *per*-*O*-acetylated β-*C*-glucopyranosyl ketone and tetrahydropyranyl vanillyl acetal under basic conditions and further cleavage of the tetrahydropyranyl and acetate protecting groups (Scheme 2). Dimer of ferulic ethyl ester **17** was obtained from the esterification of the corresponding diacid with a 95% yield, obtained in turn by a known procedure. Biphenol **1** and its derivate 3,3′-dibromo-4,4′-dihydroxy-biphenyl, were glycosylated at one phenolic–OH group by the reaction of 2,3,4,6-tetra-*O*-acetyl-α-d-glucopyranosyl bromide and K_2_CO_3_ and further hydrolysis of acetyl groups which obtained compounds **18** and **19**, respectively, as β-anomer (Scheme 3).

The oxidative coupling reaction of 3,3′-dibromo-4,4′-dihydroxy-biphenyl with linalool in the presence of [Pd(OAc)_2_] _3_ catalyst and under microwave conditions, after 10 min, led to a 40% yield of dimer **20**.

### 2.2. Spectrophotometric Detection of Tyrosinase Inhibitory Activity

The ability of compounds **7–20** to inhibit tyrosinase activity was evaluated by UV-Vis spectroscopy using either the natural substrate tyrosine or dopamine, and slightly modifying the previously described method [41,50,51]. The oxidation of tyrosine by tyrosinase enzyme induced the appearance of two distinct absorption bands at 311 and 472 nm (Figure S1). The first band was attributable to the *o*-diphenol formation, while the latter was due to the production of the *o*-quinone (Figure S1).

The time course of tyrosine oxidation at 311 nm (Figure S1 insert) showed the characteristic lag-phase due to the time required to accumulate a sufficient amount of *o*-diphenol by the catalytic activity of the small amount of oxy-tyrosinase present in the enzyme preparation. The *o*-diphenol is subsequently transformed to *o*-quinone by the *met* form of tyrosinase which is reduced to the *deoxy* form, which shows a high affinity for molecular oxygen originating the *oxy* form [21,23,52]. On the other hand, the oxidation of dopamine by tyrosinase induces the appearance of the only absorption band at about 470 nm attributable to the *o*-quinone formation (Figure S2).

As shown in the insert of Figure S2, the time course of dopamine oxidation is characterized by the absence of the lag-phase due to the *o*-diphenol structure of dopamine. This substrate, in fact, can be used to selectively evaluate the diphenolase activity of tyrosinase. In addition, a decrease in the intensity of the *o*-quinone band was detected with increasing time reaction.

**Figure 3:**
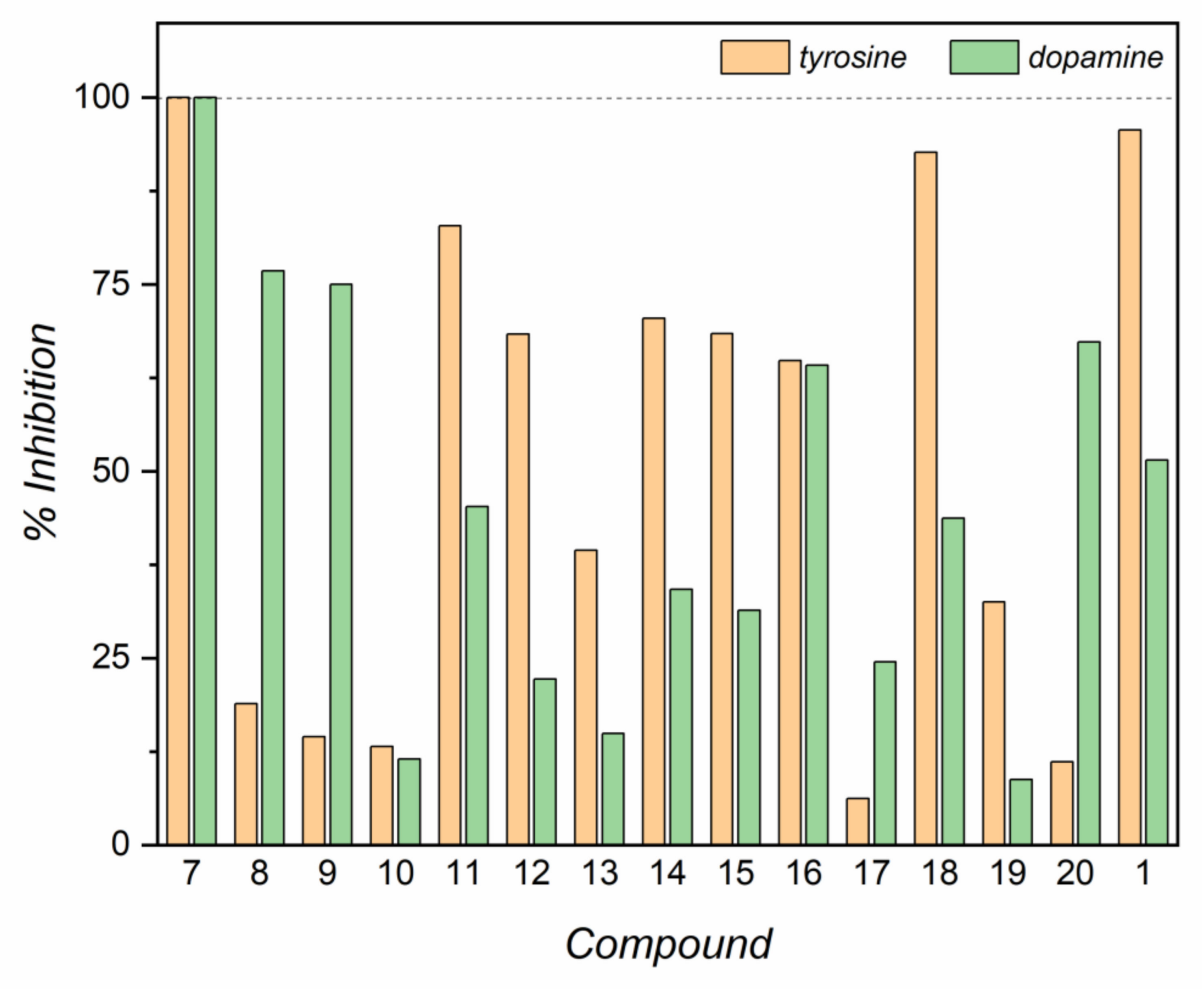
Percentage of tyrosinase inhibition determined by UV-Vis spectroscopy. Tyrosine was dissolved in 50 mM phosphate buffer, pH 6.8, final concentration 80 µM and with the added inhibitor (final concentration 20 µM) and tyrosinase (83 U/mL). The absorbance values were determined after 24 min of incubation. In the presence of dopamine, experiments were performed using 10-fold diluted solutions, and the absorbance values were determined after 10 min of incubation.

The efficiency of the tested compounds to inhibit the tyrosinase activity was determined by comparing the absorbance intensity at either 311 or 472 nm of the oxidized substrates alone or with inhibitors at different times according to Equation (1):

% inhibition = 1 − [(D − C) / (B − A)] × 100(1)

where C and D are the absorbance values at either 311 or 472 nm in presence of inhibitor before the mixing, and after either 24 or 10 min, using tyrosine or dopamine as the substrate, respectively. A and B are the corresponding values without the inhibitor. The results are summarized in Figure 3.

As shown in Figure 3, compound **7** was the most active tyrosinase inhibitor in the presence of both substrates. Similarly, compounds **16** and **10** also showed a comparable activity in both the tested substrates, although their efficiency was reduced (about 70% for **16** and only 10% for **10**). All the other tested compounds were characterized by a preferential activity in one of the used substrates. In particular, compounds **11**, **12**, **13**, **14**, **15**, **18** and **19** were characterized by a predominant inhibition of tyrosine oxidation, similar to that observed for biphenyl **1**. The remaining compounds (i.e., **8**, **9**, **17** and **20**) strongly inhibited the oxidation of dopamine compared to that of tyrosine, suggesting a selectivity towards the diphenolase activity of tyrosinase.

### 2.3. Evaluation of IC_50_ and Inhibitory Activity of Compounds **7**–**20** with Tyrosinase and Laccase Biosensors

As shown in Table 1, the IC_50_ values obtained of each compound on tyrosinase and laccase enzymes were evaluated.

For the tyrosinase enzyme, all the compounds showed IC_50_ values ranging between 20 and 130 nM. Only compound **20** showed a higher IC_50_, equal to 423 nM.

The values of IC_50_ for laccase were generally higher than those obtained for tyrosinase. Table 1 shows that compounds **7**, **11**, **13** and **14** had a lower IC_50_ value than 100 nM. Most of the compounds tested had an IC_50_ value between 118 and 218 nM. While compounds **12** and **16** yielded an IC_50_ value of about 1000 nM, compounds **9** and **18** exceeded 2000 nM.

The inhibitory activity of the compounds on tyrosinase and laccase enzymes was assessed as explained in Section 4.3. Lineweaver-Burk plots, derived from V_MAX_s and K_M_s values obtained by biosensor calibrations, whose values are shown in Table S1 in the Supplementary Materials, are reported in Figures S3–S16 and summary data are reported in Table 1. A representative IC_50_ calculation plot for tyrosinase and laccase has been reported in Figure S17 for compound **20**.

The data indicate that all the compounds are reversible inhibitors. Seven of the tested compounds produced the same type of inhibition on both enzymes, which proved to be competitive. The Lineweaver-Burk plot obtained for compound **7** highlights a competitive inhibition on the tyrosinase enzyme (Table 1), as previously demonstrated with different techniques [43]. In fact, the presence of the inhibitor resulted in a significant increase in K_M_s, but V_MAX_s was essentially the same. Only compound **14** led to a mixed inhibition on both enzymes, resulting in a significant variation in both V_MAX_ and K_M_.

The remaining compounds resulted in different types of inhibitions on the two enzymes. In most cases, such as **8**, **10** and **17**, the tyrosinase enzyme suffered from a mixed inhibition, undergoing a variation in both V_MAX_ and K_M_, while on the laccase enzyme, a competitive inhibition was highlighted for compounds **10** and **17**. On the other hand, compound **8** led to a noncompetitive inhibition, in which there was a significant variation in enzyme V_MAX,_ but not K_M_. Compound **11** led to a competitive inhibition on tyrosinase, but a mixed inhibition on laccase.

In addition, **12** and **19** produced the same type of noncompetitive inhibition on tyrosinase, as they led to a significant variation in V_MAX,_ leaving K_M_ substantially the same, but a competitive inhibition on laccase. The remaining compounds exerted different inhibition type on tyrosinase and laccase. Compound **11** produced a competitive inhibition on tyrosinase, while exerting a mixed inhibition on laccase. Finally, compound **13** produced the only uncompetitive inhibition on tyrosinase enzyme, but led to a competitive inhibition on laccase.

### 2.4. Computational Evaluation of Laccase Inhibitory Activity

**Figure 4:**
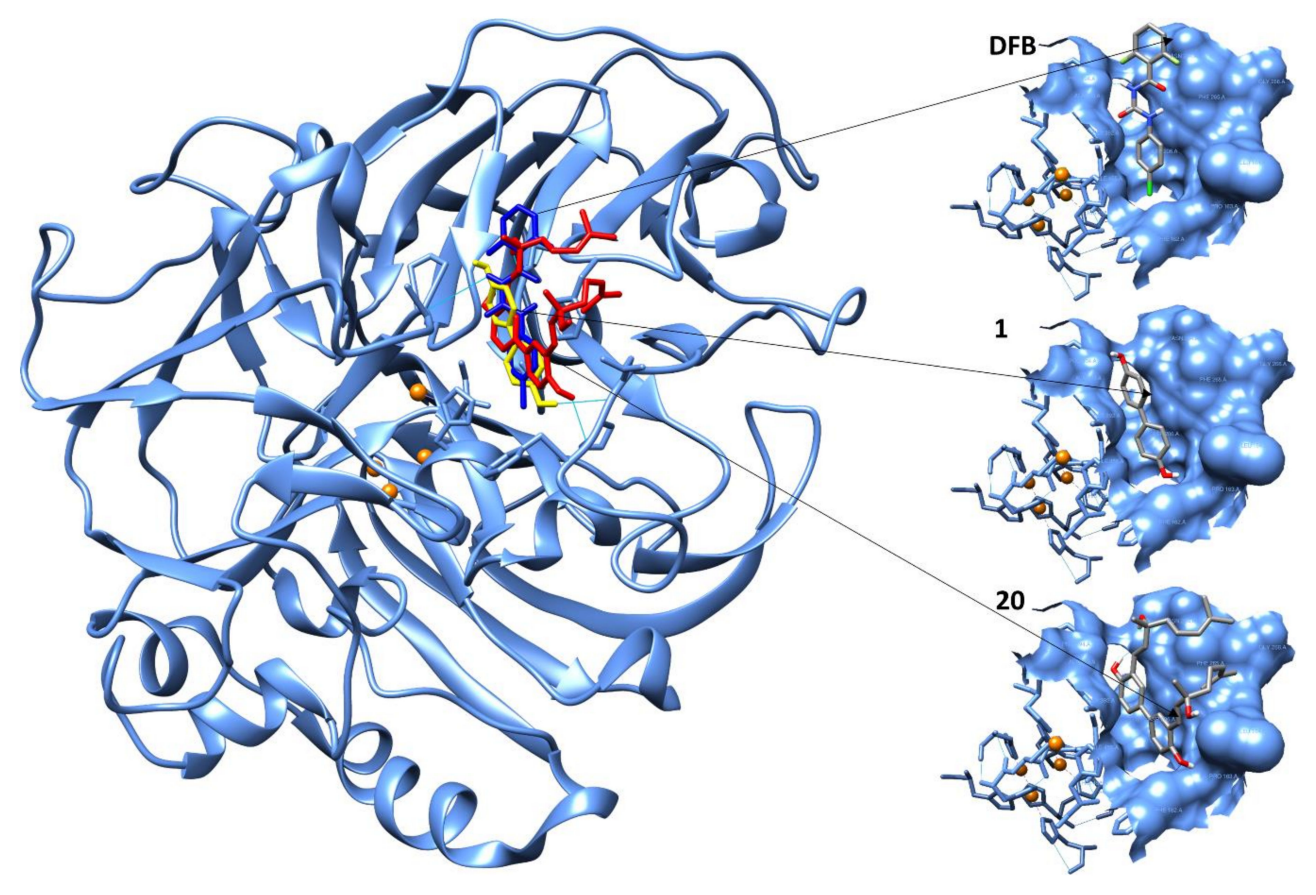
Representation of diflubenzuron (*N*-[(4-chlorophenyl)carbamoyl]-2,6-difluorobenzamide) (**DFB**), compounds **1** and **20** with the catalytic T1 site of laccase performed with Chimera [53].

The laccase X-ray structure of *Trametes versicolor* fungus [18] was used to perform molecular docking with a conventional chitin inhibitor, diflubenzuron (*N*-[(4-chlorophenyl)carbamoyl]-2,6-difluorobenzamide) (**DFB**). The best docked conformation of **DFB** (the lowest binding energy and highest population) was well embedded inside the hydrophobic binding pocket at T1 site in the crystal structure of the enzyme featured by the Cu1–N cluster formed with the residue of HIS458 (Figure 4). The chitin inhibitor fits the catalytic domain with an inhibition constant K*i* of 56.87 µM and activates H-bond with ALA393 and lipophilic interactions with HIS458 whose basic residue is involved in the key interaction Cu1–N. Table S1 lists the scores of the binding conformation for **DBF**.

**Figure 5:**
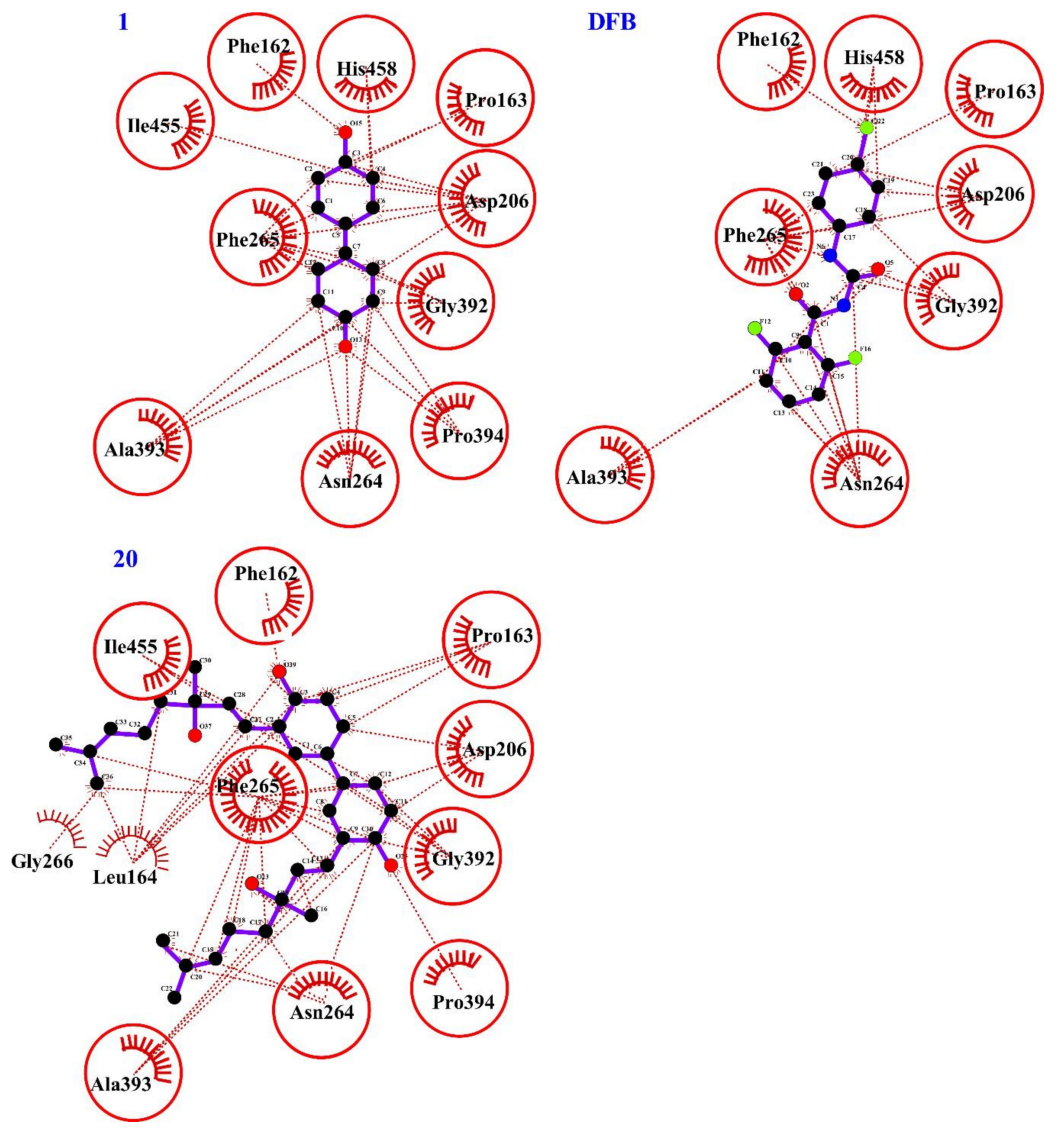
Hydrophobic interactions of **DFB**, compounds **1** and **20** with the catalytic T1 site of laccase, respectively and performed with LigPlot+ [54].

The docking protocol was evaluated then on biphenyl **1** and biphenyl **20** which both share the same hydroxylated core, but have different lipophilicity values due to two additional prenylated chains located at 3,3’ positions in biphenyl **20** (Figure 4). The interactions with the protein are almost similar to that of **DFB** and thus reveal the same lipophilic interaction with HIS458 (Figure 5 and Table S2). The K*i* values for biphenyl **1** and **20** were 108.39 and 29.54 µM, respectively. Both biphenyls activated more H-bonds than **DFB** with the following amino acids: PRO163 and ALA393 for biphenyl **1** and PHE162 and ASN264 for biphenyl **20**. Moreover, compound **20** also interacted with further amino acids involved in the catalytic site of laccase enzyme. The docking study of biphenyl **20** is in agreement with the results achieved from the kinetics study, where a competitive action was detected.

### 2.5. Cytotoxicity of Inhibitors and Protection Against Oxidative Stress

Initially, the inhibitor concentrations on PC12 cells, i.e., a rat pheochromocytoma-derived cell line, were screened to assess the possible cytotoxicity of the molecules under study [55]. As highlighted in Table 2 and in Figures S3–S16, compounds **11**, **12**, **14**, **15** and **16** were found to be toxic to cells, as they resulted in a significant (*p* < 0.05) decrease in viability, when compared with the control, ranging from a concentration of 5 μM up to 40 μM. Given the toxicity of these compounds, the protective activity against oxidative stress of these compounds was not tested.

Although the remaining compounds resulted in a significant (*p* < 0.05) decrease in viability when compared with control, they produced a loss of viability of about 20% at all the concentrations tested. There was only a more sustained decrease in viability for compounds **7**, **9**, **10** and **20**, from 30% up to 45%, but just at the highest concentrations. Given the low impact on cell viability, for these four compounds the possible protection against oxidative stress induced by hydrogen peroxide 100 μM was assessed. As shown in Figures S5–S16, most the compounds with low impact on cell viability did not protect PC12 cell culture from oxidative stress. Consequently, these compounds were unable to restore the reduced cell viability due to hydrogen peroxide. Compounds **8**, **18** and **19** produced a significant decrease (*p* < 0.05 vs H_2_O_2_) in cells viability at all concentrations tested while compounds **9** and **13** only led to a significant decrease (*p* < 0.05 vs H_2_O_2_) at a concentration of 20 μM.

Only three compounds, **7**, **10** and **20**, showed a protective activity against H_2_O_2_-induced oxidative stress, at 1 μM for compound **7**, at 20 μM for compound **10**, while at >5 μM for compound **20**, producing a significant increase (*p* < 0.05) in cells viability when compared with that produced by hydrogen peroxide. Therefore, these three compounds were also tested for protection against oxidative stress generated by manganese dichloride. As shown in Figures S3 and S6, compound **7** and **10** were not able to restore the cells viability reduced by MnCl_2_ treatment. On the contrary, compound **20** exhibited a general increase in cells viability, but which was significant (*p* < 0.05 vs MnCl_2_) only at a concentration of 1 μM.

### 2.6. Insecticidal Properties of Inhibitors

Mortality differed significantly among treatments (F_15:79_ = 44.47, *p*-value < 0.001). Of those tested, only compound **20** showed some insecticidal activity on mealworm larvae (*Tenebrio molitor*) though significantly less than diflubenzuron (**DFB**), an inhibitor of chitin synthesis (Table 3).

## 3. Discussion

Based on the design and synthesis of tyrosinase inhibitors in literature with an α,β-unsaturated carbonyl function, we focused on the synthesis of propanoids derivatives, particularly on chalcone-derivatives. We incorporated this functional motif into the structures of simple phenols and hydroxylated biphenyls (i.e., compounds **7**–**17**) and a comparison was made in terms of activity by spectrophotometric and electrochemical assays. Of the synthetic methods that provide the synthesis of an α,β-unsaturated carbonyl function conjugated to an aromatic scaffold, the Claisen-Schimdt condensation seemed to be the most straightforward route.

In compounds **7, 8** and **10**–**17** the presence of a conjugated double bond and a wide delocalization of *π*-electrons decreases the redox potential of the molecule that would be activated, as Michael acceptor, to electron transfer reactions with nucleophilic species present in the amino acids residues of the protein.

Given the role that biphenol **1** plays as tyrosinase inhibitor [41], we used this scaffold to prepare biphenyl-analogs **18**–**20** with prenylated or glycosylated units. Under pressure and microwave conditions and by palladium-catalyzed Heck reaction, we were able to reduce the time of the reaction when a prenylated chain was added to compound **1**, brominated beforehand, to achieve diprenylated biphenyl **20**.

Hydroxylated biphenyls **14**–**17** and **20** possess a C_2_-symmetry axis that create the two indistinguishable aromatic rings. This structural feature provides an increase in reaction selectivity and, in terms of molecule-protein recognition, identical interactions would take place on each symmetrical portion of the molecule. As with dimeric secondary metabolites present in plants that manifest inhibition to tyrosinase enzyme [56], we prepared hydroxylated biphenyls **14**–**20** bearing functional groups that use the chemical space effectively by increasing the structural complexity and providing more interactions with amino acid residues.

Compound **7** is a potent tyrosinase inhibitor as detected in both spectrophotometric and electrochemical assays in accordance with the literature [43]. This compound was used by us as a control. The activities of compounds **8**–**20**, all of which containing one or two phenolic–OH groups, were correlated to **7**. Spectrophotometric results showed that in the chalcone series the presence of electron donating groups in both aromatic rings promoted enzyme–inhibition interactions (i.e., compound **10** versus **11**, **12** and **14**–**16**, Figure 3). The presence of a phenolic–OH group in 4-position of the aromatic ring plays a key role in the activity of chalcones monomers **11** and **12**, highlighting that **11** has a higher percentage of inhibition given the better electron delocalization throughout the structure.

Unlike monomers **11** and **12**, the position of the phenolic–OH group in the A or B ring of chalcone structure was irrelevant in the corresponding dimers **14** and **15.** This is likely due to the extended electron delocalization in the biphenylic structure and the improved π-π stacking interactions that contributed to effective tyrosinase inhibition. Similar inhibitory activity to the monophenolasic activity was detected for dimer **16** where the phenolic–OH group is in ortho-position thus creating a six-membered intramolecular H-bonded ring which would stabilize the compound and promote the copper coordination of the tyrosinase catalytic domain. A hydroxylated biphenyl structure was also used as a lead structure for the synthesis of compounds **17**–**20** where a different trend of mono and diphenolasic activity was detected in relation to the moiety bonded to the hydroxylated core. Free phenolic–OH group and high hydrophilicity (i.e.*,* compound **18**) both favored monophenolasic activity whereas the increase of lipophylicity as in compounds **19** and **20** affected that enzyme phase.

The diphenolase activity of tyrosinase was determined using dopamine as substrate using the spectrophotometric method. The decrease in the intensity of the *o*-quinone band (Figure S2) was likely due to the reaction of the *o*-quinone moiety with either the amino group of dopamine (intramolecular reaction) or reactive groups (amino, hydroxyl, thiol) present on the surface of the enzyme. This behavior has been described for analogs of caffeic acid, which at high concentrations act as irreversible (suicide) inhibitors of tyrosinase due to the high reactivity of the corresponding *o*-quinone [57,58]. Compounds **8**, **9**, **17** and **20** manifested a selectivity towards the diphenolasic phase probably due to a restricted molecular volume, high structural flexibility and antioxidant activity which together may provide strong interactions with the amino acids residues or with the substrate dopamine.

Electrochemical detection of dopamine oxidation in the presence of the studied compounds performed by biosensors and based on the immobilization of tyrosinase and laccase enzymes provided useful information on the diphenolasic phase by identifying the type of interaction. In a biosensor, the mechanism of enzyme inhibition is often complex due to the enzyme immobilization, thus the interference with the active site by the action of an inhibitor could give different results from those achieved when the enzyme is in a free aqueous medium as in the spectrophotometric assay.

The mode of inhibition can be competitive, uncompetitive, mixed type (competitive/uncompetitive) and noncompetitive. A competitive inhibitor can bind to the free enzyme preventing the substrate from binding to the catalytic active site, whereas an uncompetitive inhibitor binds only the enzyme–substrate complex. When a compound binds to the free enzyme and the enzyme–substrate complex with the same equilibrium constant, it can be considered a noncompetitive inhibitor. Mixed and competitive types are the main modes of inhibition observed for phenols and polyphenols in kinetics studies on mushroom tyrosinase [23].

Even though to different extent, compounds **7**, **9**, **15**, **16**, **18** and **20** inhibited both tyrosinase and laccase enzymes competitively by mimicking the substrate of the enzyme and, probably, by trapping the *o*-dopaquinone intermediate due to the nucleophilic feature of the phenolic–OH group present in these compounds. Compounds **10**, **13** and **17** are uncompetitive or mixed inhibitors for tyrosinase enzyme by binding the enzyme-dopamine complex, whereas they showed a competitive inhibition in the presence of laccase. Laccases tend to have a broader range of substrates than that of tyrosinase. Our data confirmed this trend by identifying eleven compounds as competitive inhibitors for laccase (Table 1). Interestingly, a different behavior was observed between chalcones isomers **11** and **12** towards tyrosinase and laccase, respectively, highlighting how a different electron delocalization and a strong hydrogen bond may influence the interactions enzyme-substrate-inhibitor.

With regard to IC_50_, all the compounds studied, except for compound **20**, had low value in the tyrosinase series, whereas laccase had a broader range of the IC_50_ values and was sensitive to the compound structure. Since conventional inhibitors suffer from toxicity and lack of efficacy, all compounds were subjected to viability assay on PC12 cells. Compounds **17**–**20** showed almost no toxicity (cell viability −80%) on PC12 cells up to 40 µM and among them, biphenyl **20** protected against oxidative stress caused by H_2_O_2_ and MnCl_2_ treatments.

Taking in account the key role that different types of laccases have in the cuticle of many insects species suggesting an involvement in cuticle sclerotization, compound **20** together with compounds **7**-**9**, **11**, **12**, **14** and **15** which showed a good inhibition to laccase enzyme, were assayed in vivo against larvae of *Tenebrio molitor* as a model insect. Tyrosinase inhibitors **1**–**6**, which we had detected in the diphenolasic phase [41], were added to the same assay. Of the compounds tested, only biphenyl **20** was effective against mealworm larvae of *Tenebrio molitor* that ranged about 65% of mortality in comparison to **DFB**, a conventional chitin inhibitor. The molecular docking studies carried on the crystal structure of *Trametes versicolor* fungus [18] showed that compounds **20**, **1** and **DFB** fit the catalytic domain almost with the same cluster of amino acids. Compared to biphenol **1** and **DFB**, compound **20** activated stronger interactions (H-bond) especially with PHE162 and ASN264. Interestingly, the lowest inhibition constant was estimated for compound **20** (K*i* 29.54 µM), probably due to an acquired conformational flexibility of the structure bearing a hydroxylated biphenyl and two identical hydroxyprenylated chains that activate a greater number of interactions, which are also more specific, with the amino acids residues. Interactions of compound **20** with HIS458, which is a putative key amino acid in the Cu1–N bond at T1 site [18], may affect the hypothesized contribution of the free electron pair from the N to the Cu1, rendering the protein less active. The fact that biphenyl **20** showed the best affinity for the catalytic site of laccase protein (lowest K*i* value) is in agreement with results achieved from a spectrophotometric assay that highlighted a more inhibitory effect of **20** in the diphenolasic activity than with biphenyl **1**, and this was further confirmed by an in vivo experiment against larvae of *Tenebrio molitor*. In addition. the influence of the prenylated chain of compound **20** in the mortality of the mealworm larvae measured in in vivo assay in comparison with the mortality caused by hydroxylated biphenyls **1**–**6** and **14**–**15** cannot be ruled out. In fact, a prenylated moiety interferes with the lipophilic proteins embedded in the outer membrane of microorganisms and insects leading to destruction of the cuticle and favoring intracellular uptake of the inhibitor [59].

## 4. Materials and Methods

### 4.1. Synthesis of Compounds ***18*** and ***19*** from Derivates ***21*** and ***22***

#### General Procedure for the Synthesis of **21** and **22**

To a solution of 4,4′-dihydroxy-biphenyl **1** or 3,3ʹ-dibromo-4,4′-dihydroxy-biphenyl [60] (1 eq) in anhydrous acetone (100 mL), K_2_CO_3_ (1,5 eq) and 2,3,4,6-tetra-O-acetyl-α-d-glucopyranosyl bromide (1,5 eq mmol) were added. The mixture was stirred for 48 h at 50 °C. The solvent was evaporated in vacuum, the crude product was diluted with diethyl ether, acidified with hydrochloridric acid (10% solution) and extracted with diethyl ether (3 × 50 mL), the organic phases were combined and dried over anhydrous sodium sulfate. The crude product was purified by flash chromatography using a 1:1 mixture of petroleum ether:ethyl acetate (for **21**) or 2:1 mixture of petroleum ether: ethyl acetate (for **22**) as eluent to give compound **21** or **22** as white solid.

**(2*R*,3*R*,4*S*,5*R*,6*S*)-2-(acetoxymethyl)-6-((4’-hydroxy-[1,1’-biphenyl]-4-yl)oxy)tetrahydro-2*H*-pyran-3,4,5-triyl triacetate 21**: (25%): mp = 83–84 °C; [α] _D_^20^ 18.8 (c = 1.0, CHCl_3_); ^1^H-NMR (CD_3_COCD_3_) δ 1.96 (s, 3H), 2.02 (s, 3H), 2.17 (s, 3H), 2.27 (s, 3H), 4.21 (d, *J* = 6.8 Hz, 2H), 4.46 (td, *J* = 0.8, 6.4 Hz, 1H), 5.28 (m, 1H), 5.43–5.49 (series of m, 3H), 7.15–7.20 (series of m, Ar, 4H), 7.60–7.64 (series of m, Ar, 4H); ^13^C-NMR (CD_3_COCD_3_) δ 19.70, 19.72, 19.81, 20.17, 67.38, 68.64, 70.71, 70.92, 71.02, 117.06, 122.17, 127.52, 128.05, 134.91, 137.80, 150.34, 156.80, 168.91, 169.46, 169.57, 169.75, 169.97. Anal. Calcd. for C_26_H_28_O_11_ C, 60.46; H, 5.46 Found: C, 60.52; H, 5.48.

**(2*R*,3*R*,4*S*,5*R*,6*S*)-2-(acetoxymethyl)-6-(3,3’-dibromo-4’-hydroxy-[1,1’-biphenyl]-4-yl)oxy)tetrahydro-2*H*-pyran-3,4,5-triyl triacetate 22:** (26%): mp = 109–110 °C; [α] _D_^20^ 10.2 (c = 1.0, CHCl_3_); ^1^H-NMR δ 2.04 (s, 3H), 2.08 (s, 3H), 2.12 (s, 3H), 2.20 (s, 3H), 4.10 (t, *J* = 8.4 Hz, 1H,), 4.20 (dd, *J* = 6.0, 11.2 Hz, 1H), 4.26 (dd, *J* = 6.8, 11.2 Hz, 1H,); 5.01 (d, *J* = 7.6 Hz; 1H), 5.13 (dd, *J* = 3.6, 10.8 Hz; 1H), 5,4 (d, *J* = 3.2 Hz, 1H), 5.61(dd, *J* = 8.4, 10.8 Hz; 1H), 7.17 (d, *J* = 8.0 Hz, Ar, 1H,), 7.21 (d, *J* = 8.4 Hz, Ar, 1H), 7.39 (dd, *J* = 2.4, 8.8 Hz, Ar, 1H); 7.45 (dd, *J* = 2.0, 8.8, Ar, 1H), 7.71 (d, *J* = 2.0 Hz, Ar, 1H); 7.74 (d, *J* = 2.0 Hz, Ar, 1H); ^13^C-NMR δ 20.61, 20.68, 20.71, 20.81, 66.79, 68.07, 70.66, 71.28, 100.53, 113.70, 116.73, 117.91, 124.03, 126.98, 127.13, 131.66, 132.02, 135.78, 138.67, 147.77, 153.44, 168.32, 169.35, 170.16, 170.23. Anal. Calcd. for C_26_H_26_Br_2_O_11_ C, 46.31; H, 3.89 Found: C, 46.52; H, 3.94.

#### General Procedure for the Synthesis of **18** and **19**

To a solution of compound **21** or **22** (1 eq) in MeOH:EtOAc 1:1 solution (16 mL), sodium methoxide (1.45 eq) was added. The solution was stirred at room temperature for 1 h. The reaction mixture was neutralized using a Dowex Marathon C (H^+^ form) resin for 10 min at room temperature, filtrated and concentrated in vacuo to obtain **18** or **19**.

**(2*S*,3*R*,4*S*,5*S*,6*R*)-2-((4’-hydroxy-[1,1’-biphenyl]-4-yl)oxy)-6-(hydroxymethyl)tetrahydro-2*H*-pyran-3,4,5-triol 18:** white solid (85%): mp = 260–262 °C; [α] _D_^20^ −22 (c = 0.3, MeOH); ^1^H-NMR (CD_3_OD) δ 3.57 (dd, *J* = 3.6, 10.0 Hz, 1H), 3.69–3.90 (series of m, 4H), 3.91 (d, *J* = 2.4 Hz 1H), 4.86 (d, *J* = 10.0 Hz, 1H), 6.80 (m, Ar, 2H); 7.12 (m, Ar, 2H); 7.38 (m, Ar, 2H); 7.44 (m, Ar, 2H); ^13^C-NMR (CD_3_OD) δ 60.99, 68.80, 70.88, 73.44, 75.54, 101.60, 115.13, 116.64, 126.89, 127.28, 132.03, 135.27, 145.34, 156.66. Anal. Calcd. for C_18_H_20_O_7_ C, 62.06; H, 5.79 Found: C, 62.50; H, 5.58.

**(2*S*,3*R*,4*S*,5*S*,6*R*)-2-((3,3’-dibromo-4’-hydroxy-[1,1’-biphenyl]-4-yl)oxy)-6-(hydroxymethyl)tetrahydro-2*H*-pyran-3,4,5-triol 19**: white solid (85%): mp 176–178 °C; [α] _D_^20^ 19.8 (c = 0.3, MeOH); ^1^H-NMR (CD_3_COCD_3_) δ 3.88–3.99 (series of m, 4H), 4.17 (d, *J* = 5.6 Hz 1H), 4.86 (d, *J* = 4.0 Hz, 1H), 5.04 (d, *J* = 7.6 Hz, 1H), 7.06 (d, *J* = 8.8 Hz, Ar, 1H), 7.33 (d, *J* = 8.4 Hz, Ar, 1H); 7.48 (dd, *J* = 2.4, 8.4 Hz, Ar, 1H), 7.52 (dd, *J* = 2.4, 8.8 Hz, Ar, 1H,); 7.76 (d, *J* = 7.6 Hz, Ar, 1H); 7.78 (d, *J* = 2.4 Hz, Ar, 1H); ^13^C-NMR (CD_3_COCD_3_) δ 61.39, 68.88, 71.12, 73.85, 75.82, 101.65, 110.06, 112.37, 116.64, 116.75, 126.58, 126.96, 130.75, 130.95, 132.31, 134.59, 153.44, 153.58. Anal. Calcd. for C_18_H_18_Br_2_O_7_: C, 42.71; H, 3.58 Found: C, 42.93; H, 3.62.

**3,3’-bis((*E*)-3-hydroxy-3,7-dimethylocta-1,6-dien-1-yl)-[1,1’-biphenyl]-4,4’-diol 20.**

To a solution of 3,3ʹ-dibromo-4,4ʹ-dihydroxybiphenyl [60] (1.00 g, 2.91 mmol) in DMF (10 mL), Cs_2_CO_3_ (0.94 g 2.91 mmol), [Pd(OAc)_2_] _3_ (10% mol, 0.19 g, 0.29 mmol) and linalool (1.79 g, 11.64 mmol) were added. The reaction mixture was stirred under MW irradiation for 10 min at 100 °C. The crude product was diluted with Et_2_O and finally washed with brine. The organic layer was dried over anhydrous sodium sulfate, concentrated under vacuum and purified by flash chromatography, using a 3:1 mixture of Et_2_O:petroleum ether as eluent to give **20** as brown solid (0.57 g, 40%): mp = 127–128 °C; ^1^H-NMR (CD_3_COCD_3_) δ 1.37 (s, 6H); 1.58 (s, 6H),1.61–1.65 (series of m, 4H), 1.63 (s, 6H); 2.04–2.21 (series of m, 4H); 5.15 (m, 4H), 6.47 (d, *J* = 16.4 Hz, 2H), 6.91 (d, *J* = 8 Hz, Ar, Ar, 2H), 6.98 (d, *J* = 16.4 Hz, 2H), 7.28 (dd, *J* = 2.4 Hz, 8.0 Hz, Ar, 2H); 7.64 (d, *J* = 2.4 Hz, Ar, 2H); 8.51(bs, 2 OH); ^13^C-NMR (CD_3_COCD_3_) δ 16.84, 22.79, 24.98, 27.97, 43.06, 72.22, 115.96, 121.52, 124.67, 124.76, 125.00, 126.02, 130.48, 132.82, 137.67, 153.57. Anal. Calcd. for C_32_H_42_O_4_: C, 78.33; H, 8.63; Found: C, 78.41; H, 8.68.

### 4.2. Spectrophotometric Assay

Kinetic assays were carried out by measuring the appearance of the product of either tyrosine or dopamine oxidation in the reaction medium at 311 and 472 nm, respectively, in a Shimadzu UV-2501 spectrophotometer (Shimadzu, Kyoto, Japan) equipped with the UV-Probe software, using a tandem quartz cuvette (Hellma, Milan, Italy) with a 2 × 4.375-mm path length [1,2,51]. Briefly, one side of the dual chamber cuvette was filled with 1000 µl of mushroom tyrosinase solution in 50 mM phosphate buffer, pH 6.8, 166 Units/mL using tyrosine as substrate or a 16.6 Units/mL when dopamine was used as substrate. The other chamber was loaded with 980 µL of substrate solution (180 µM tyrosine or 18 µM dopamine) in 20 mM phosphate buffer (pH 6.8) added of either 20 µL of DMSO (control) or 20 µL of a 2 mM (0.2 mM in dopamine experiments) solution of tested inhibitors in DMSO. A background spectrum in the 250–700 nm range was acquired with a 2.0 nm slit and fast speed before starting the reaction. Thereafter, the solutions in the two chambers were mixed by inversion and the UV-Vis spectra were acquired at increasing time intervals. Spectra were recorded at 311 or 472 nm in the presence of the inhibitor before mixing, and after either 24 or 10 min, using tyrosine or dopamine as a substrate, respectively. Data processing was performed with UV Probe and OriginPro 2019 (v. 9.60) softwares.

The mushroom tyrosinase was used as purchased without further purification. All solutions (tyrosinase, substrates and inhibitors) were filtered through a 0.45-μm syringe filter, aliquoted in Eppendorf tubes, and stored in freezer. The relative concentration was determined spectrophotometrically (ε_tyrosine_ 274.6 nm = 1420 M^−1^·cm^−1^; ε_dopamine_ at 280 nm = 2670 M^−1^·cm^−1^; ε_tyrosinase_ at 280 nm = 1426 M^−1^·cm^−1^).

### 4.3. Inhibition Protocols on Tyrosinase and Laccase Biosensors

Inhibition types and IC_50_ were assessed by means of carbon-based amperometric biosensors [61,62,63,64,65], that are described in the Supplementary Materials. During the experiments carried out to determine the IC_50_ values, the lowest inhibitory concentration of each compound was also evaluated.

To verify the inhibition types, the following protocol was used. An initial calibration with dopamine, ranging between 0 and 140 mM, was performed in order to ascertain the values of V_MAX_ and K_M_. Another calibration with the same protocol was performed after having exposed the biosensors to twice the lowest inhibiting concentration of each inhibitor, in order to ensure the safe inhibition of enzymatic activity. The biosensors were exposed to the inhibitor for 30 min and after having reached a stabile baseline, a calibration with dopamine, ranging between 0 and 140 mM, was performed and the value of V_MAX_ and K_M_ were calculated. The variations of both V_MAX_ and K_M_ were then analyzed by using the Lineweaver-Burk plot in order to identify the type of inhibition induced by the tested compound. The statistical difference in V_MAX_ and K_M_, before exposing the biosensors to the inhibitor and then afterwards, was calculated by means of a paired *t-*test (*p* < 0.05).

### 4.4. Computational Equipment and Software

The crystal structure of a laccase from the fungus *Trametes Versicolor* was released from the RCSB protein databank (PDB ID: 1GYC) (www.rcsb.org) [18]. Computational modelling experiments were carried out on an HP8100 PC and an EXXACT Tensor Workstation TWS-1686525-AMB with OS Ubuntu 18.4 or Windows 10 and Centos, respectively. The atomic charges were assigned using the Gasteiger-Marsili method for the ligand and the protein [66]. Binding of the compounds was analyzed using MGLTools 1.5.7rc1 [67] and AutoDock 4.2.6 docking programs [68,69]. The structures were docked using the Lamarckian genetic algorithm (LGA). In all the docking procedures, the LGA was defined through a centerd grid, coordinates: x = 10, y = 20, z = 38, with 60 × 60 × 60 grid points in x, y, z dimensions, respectively, spacing 0.375 Å. The Lamarckian genetic algorithm (LGA) with up to 100 runs was set to the population size of 150 individuals, maximum number of generations and energy evaluations of 27,000 and 25,000,000, respectively. From the estimated free energy of ligand binding (E.F.E.B., ΔG), the estimated inhibition constant (E.I.C., K*i*) for each ligand was calculated. K*i* is calculated by the Equation (2):

K*i* = exp[(ΔG × 1000)/(R × T)] (2)

where ΔG is the docking estimated free energy, R (gas constant) = 1.98719 cal/(K × mol) and T = 298.15 K. The poses derived from the docking were represented graphically using Chimera v.1.13.1 [53]. **DFB**, **1** and **20** structures were built using the standard fragment database as in MacroModel v.6.0 [70]. AMBER force field was used for the minimization of structures, performed with MacroModel/BachMin v.6.0. An extensive conformational search was carried out using the Monte Carlo/energy minimization [71]. Representative minimum energy conformations of each compound were optimized using density functional theory (DFT) quantum chemistry program Gaussian 09 W (DFT, B3LYP, 6–311G) (Wallingford, CT, USA). Visual analysis was performed with GaussView v.5.0 [72,73].

### 4.5. Testing Insecticidal Properties on Mealworm Larvae

Late instar larvae of *Tenebrio molitor* from a twenty-year-old insect culture at the Research Institute on Terrestrial Ecosystems (National Research Council, Sassari, Italy) were used in the experiments. The species was reared on bran flour, with the integration of fresh vegetables, at 25 °C, 65% relative humidity and 16:8 hours light/darkness.

One hundred medium instar larvae were isolated in a petri dishes (diam. 50 mm). They were then immersed for three seconds in 2.5 mL of an acetone solution (concentration 6.4 mg/mL *w:v*) of one of the fourteen compounds listed in Table 3. In addition, a treatment with the commercial formulation Dimilin (WP, 25% a.i. diflubenzuron) (acetone solution, 25.6 mg/mL *w:v*) and a negative control (no treatment) were tested.

After treatment, the larvae were subdivided into five 20-specimen groups and reared for five days in a small PVC box (150 L × 120 W × 50 H mm) with the same feed and conditions as standard rearing. Mortality was recorded daily.

The statistical analysis was conducted with the R software (R Development Core Team 2016). Mortality data, expressed as a percentage, were arcsin(√x)-transformed to satisfy assumptions of normality. ANOVA was performed by the Duncan test for mean separation.

## 5. Conclusions

Novel multitarget tyrosinase and laccase enzymes inhibitors featured by a phenylpropanoid and hydroxylated biphenyl core were prepared under straightforward synthetic procedures starting from naturally occurring, commercially available compounds. A drug repositioning concept was applied to discover effective tyrosinase and laccase inhibitors. Both spectrometric and electrochemical assays enabled us to reveal the inhibitory activity of the compounds identifying, by PC12 cell viability, the structure of safe candidates for the future. In silico studies performed on the crystal structure of the laccase enzyme and in vivo experiments identified a promising lead structure in compound **20** for the control and management of many species of insects.

## Figures and Tables

**Table 1:** Calculated IC_50_ of each compound on tyrosinase and laccase enzymes and type of inhibition exerted. Concentrations are expressed in nM.

Compound	IC_50_		Type of Inhibition	
	Tyrosinase	Laccase	Tyrosinase	Laccase
**7**	26	27	competitive	competitive
**8**	54	218	mixed	noncompetitive
**9**	122	2238	competitive	competitive
**10**	101	212	mixed	competitive
**11**	38	62	competitive	mixed
**12**	72	1043	noncompetitive	competitive
**13**	20	23	uncompetitive	competitive
**14**	22	47	mixed	mixed
**15**	120	209	competitive	competitive
**16**	62	987	competitive	competitive
**17**	130	184	mixed	competitive
**18**	65	2619	competitive	competitive
**19**	24	130	noncompetitive	competitive
**20**	423	118	competitive	competitive

**Table 2:** Results obtained on PC12 cells following the screening of concentrations (from 1 to 40 μM) of the compounds under study, the co-treatment with 100 μM H_2_O_2_ and with 1 mM MnCl_2_.

Compound	Toxic Concentration (µM) ^1^	H_2_O_2_ Damage Protection (µM)	MnCl_2_ Damage Protection (µM)
**7**	>10	1	n.p.
**8**	40	n.p.	n.a.
**9**	>10	n.p.	n.a.
**10**	>20	20	n.p.
**11**	>10	n.a.	n.a.
**12**	>1	n.a.	n.a.
**13**	>20	n.p.	n.a.
**14**	>5	n.a.	n.a.
**15**	>5	n.a.	n.a.
**16**	>5	n.a.	n.a.
**17**	none	n.p.	n.a.
**18**	none	n.p.	n.a.
**19**	none	n.p.	n.a.
**20**	none	>5	1

^1^ cell viability < 80%; n.p.: no protection; n.a.: not available;

**Table 3:** Insecticide activity, as percentage of mortality, on mealworm larvae (*Tenebrio molitor*) of fourteen of inhibitors compared with DFB^*^.

Compounds	Mortality% (Mean ± SD)**	Arcsine(√x) Transformation	Mean Comparison*** (Duncan Test, α = 0.05)				
**DFB**	97.0 ± 4.47	1.46 ± 0.15	a				
control	1.0 ± 2.24	0.05 ± 0.10					e
**1**	2.0 ± 2.74	0.09 ± 0.12					e
**2**	4.0 ± 5.48	0.13 ± 0.18				d	e
**3**	3.0 ± 2.74	0.14 ± 0.12				d	e
**4**	3.0 ± 4.47	0.11 ± 0.15				d	e
**5**	16.0 ± 9.62	0.40 ± 0.13			c		
**6**	1.0 ± 2.24	0.05 ± 0.10					e
**7**	5.0 ± 7.07	0.14 ± 0.20				d	e
**8**	10.0 ± 7.07	0.29 ± 0.17			c	d	
**9**	4.0 ± 6.51	0.12 ± 0.18				d	e
**11**	4.0 ± 2.24	0.18 ± 0.10				d	e
**12**	0	0					e
**14**	0	0					e
**15**	0	0					e
**20**	67.0 ± 14.83	0.97 ± 0.16		b			

* Treatments for immersion in 2.5 mL compound/acetone solution at concentration of 6.4 mg/mL (*w:v*). Details in Section 4.5. **Mean ± SD of mortality proportion data after arcsine transformation (arcsine of the square root). ***Means followed by the same letter are not significantly different.

## Supplementary Materials

The following are available online, 1.1. Chemistry—synthesis of compounds **7** and **12**–**17**; 1.2. Spectrophotometric assay of tyrosine and dopamine, Figures S1 and S2; 1.3. Electrochemical assays: biosensor calibration and inhibition protocols; 1.4. Viability and oxidative stress assays (Table S1. Kinetic parameters of calibrations); 1.5. Lineweaver-Burk plot for compounds **7** (Figure S3), **8** (Figure S4), **9** (Figure S5), **10** (Figure S6), **11** (Figure S7), **12** (Figure S8), **13** (Figure S9), **14** (Figure S10), **15** (Figure S11), **16** (Figure S12), **17** (Figure S13), **18** (Figure S14), **19** (Figure S15) and **20** (Figure S16) on tyrosinase and laccase enzymes; 1.6. Representative plot of IC_50_ calculation for compound **20**, (Figure S17); 1.7. Computational studies of compounds DFB, **1** and **20** (Tables S2 and S3).
