# Supplementary material for: Synthesis and Studies of the Inhibitory Effect of Hydroxylated Phenylpropanoids and Biphenols Derivatives on Tyrosinase and Laccase Enzymes
Source: Molecules. 2020 Jun 11;25(11):2709. doi: 10.3390/molecules25112709 (PMC7321210; doi:10.3390/molecules25112709)
Supplement: Supplementary file 1 [file molecules-25-02709-s001.pdf]

Supplementary material for:

Synthesis and studies of the inhibitory effect of hydroxylated phenylpropanoids and biphenols derivatives on tyrosinase and laccase enzymes

Maria Antonietta Dettori[1,#], Davide Fabbri[1,#], Alessandro Dessì[1], Roberto Dallocchio[1], Paola Carta[1], Claudia Honisch[2,3], Paolo Ruzza[2,3], Donatella Farina[4], Rossana Migheli[4], Pier Andrea Serra[4], Roberto A. Pantaleoni[5,6], Xenia Fois[6], Gaia Rocchitta[4,*], Giovanna Delogu[1,*]

1 Istituto di Chimica Biomolecolare, Consiglio Nazionale Ricerche, Sassari, Italy. M.A.D. mariaantonietta.dettori@cnr.it; D.F. davidegaetano.fabbri@cnr.it; A.D. alessandro.dessi@cnr.it; R.D. robertonico.dallocchio@cnr.it; P.C. paola.carta@cnr.it; G.D. giovanna.delogu@icb.cnr.it

2 Dipartimento di Scienze Chimiche, Università degli Studi di Padova, Padova, Italy. C.H. claudia.honisch@phd.unipd.it

3 Istituto di Chimica Biomolecolare, Consiglio Nazionale Ricerche, Padova, Italy. C.H. claudia.honisch@unipd.it; P.R. paolo.ruzza@cnr.it.

4 Dipartimento di Scienze Mediche, Chirurgiche e Sperimentali, Università degli Studi, Sassari, Italy. D.F. donatellafarina@tiscali.it; R.M. rmigheli@uniss.it; P.A.S. paserra@uniss.it; G.R. grocchitta@uniss.it

5 Istituto di Ricerca sugli Ecosistemi Terrestri, Consiglio Nazionale Ricerca, Sassari, Italy. R.P. roberto.pantaleoni@iret.cnr.it; X.F. xeniafois@hotmail.it

6 Dipartimento di Agraria, Università degli Studi, Sassari, Italy. R.A.P. pantaleo@uniss.it;

# M.A.D. and D.F. contributed equally to this work.

* Correspondence: +39 079228526, grocchitta@uniss.it (G.R.); +39 079 2841220, giovanna.delogu@icb.cnr.it (G.D.)

Table of the contents

1.1 Chemistry synthesis of compounds **7** and **12-17** S2

1.2 Spectrophotometric assay of tyrosine and dopamine: Figures S1 and S2 S5

1.3 Electrochemical assays: biosensor calibration and inhibition protocols S6

1.4 Viability and oxidative stress assays S7

1.5 Kinetic parameters, Lineweaver–Burk plot and viability graphics of compounds **7-20:** Table S1 and Figures S3-S16 S8

1.6 Representative plot of IC_{50} calculation for compound **20** S23

1.7 Computational studies of compounds **DFB**, **1** and **20**: Tables S2 and S3 S24

1.8 References S25

1.1 Chemistry

Unless otherwise noted, starting materials and reagents were obtained from commercial suppliers and were used without further purification. Melting points were determined on a Büchi 530 apparatus and are uncorrected. All ^{1}H NMR and ^{13}C NMR spectra were recorded in $CDCl_3$ (if not otherwise indicated) solution with a Varian VXR 5000 spectrometer at 399.94 MHz and 75.42 MHz respectively. Chemical shifts are given in ppm (δ); multiplicities are indicated by s (singlet), d (doublet), t (triplet), q (quartet), m (multiplet), td (triplet of doublets) or dd (doublet of doublets). Elemental analyses were performed using an elemental analyser Perkin-Elmer model 240 C. Acetone was freshly distilled from $CaCl_2$. Flash chromatography was carried out with silica gel 60 (230-400 mesh, Kiesgel, EM Reagents) eluting with appropriate solution in the stated v:v proportions. Analytical thin-layer chromatography (TLC) was performed with 0.25 mm thick silica gel plates (Polygram® Sil G/UV254, Macherey-Nagel). All reactions were monitored by TLC performed on 0.2 mm thick silica gel plates (60 F254 Merck). The purity of all new compounds was judged to be >98% by ^{1}H NMR spectral determination.

Compounds **8** *(E)-4-(4-hydroxy-3-methoxyphenyl)but-3-en-2-one)* , **9** *4-(3,4-dihydroxyphenyl)butan-2-one*, **10** *(E)-3-(4-hydroxy-3-methoxyphenyl)-1-phenylprop-2-en-1-one* and **11** *(E)-1-(3,4-dimethoxyphenyl)-3-(4-hydroxy-3-methoxyphenyl)prop-2-en-1-one* were prepared according to the literature procedures [1-4].

(E)-4-(2,4-dihydroxyphenyl)but-3-en-2-one **7**

To a stirred solution of 2,4-dihydroxybenzaldehyde (3.0 g, 21.7 mmol) in acetone (50 mL) at room temperature and under N_2, an aqueous solution (1 N) of NaOH (80 mL, 80 mmol) was added dropwise. The mixture was stirred at 50°C for 12 h. The solvent was roto-evaporated, water and hydrochloridric acid (10% solution) were cautiously added. The heterogeneous solution was extracted with ether, dried over anhydrous sodium sulphate and evaporated. The crude product was purified by flash chromatography using a 1: 1 mixture of petroleum ether: ethyl acetate as eluent to give compound **7** as dark solid (2.3 g, 60%): mp 119-121°C (lit.[5] 118-120 °C); ^{1}H-NMR (CD_3COCD_3) δ 2.25 (s, 3H), 6.41 (dd, *J* = 2.0, 8.4 Hz, Ar, 1H), 6.47 (d, *J* = 2.0 Hz, Ar, 1H), 6.67 (d, *J* = 16.4 Hz, 1H), 7.48 (d, *J* = 8.4 Hz, Ar. 1H), 7.82 (d, *J* = 16.4 Hz, Ar, 1H); ^{13}C-NMR (CD_3COCD_3) δ 26.34, 102.69, 108.14, 113.73, 123.88, 129.95, 138.41, 158.32, 160.92, 197.07. Anal. Calcd. for $C_{10}H_{12}O_3$: C, 66.65; H, 6.71 Found: C, 66.72; H, 6.53.

(E)-1-(3,4-dimethoxyphenyl)-3-(4-hydroxy-3-methoxyphenyl)prop-2-en-1-one **12**.

To a stirred solution of KOH (1.14 g, 20.32 mmol) in water (4 mL) was added dropwise a solution of 3,4-dimethoxybenzaldehyde (1.70 g, 10.23 mmol) and apocynin (1.70 g, 10.23 mmol) in methanol (20 mL). The reaction mixture was stirred at room temperature for 96 h under N_2. The mixture was poured into ice–water (10 mL), adjusted to pH 3–4 with hydrochloridric acid (10% solution), and then extracted with ethyl acetate. The organic layer was successively washed with water and saturated brine, dried over anhydrous sodium sulphate and purified by flash chromatography using a 2:1 mixture of ethyl acetate:petroleum as eluent to give **12** as a yellow solid (2.72 g, 85%): mp = 110-112°C; ^{1}H NMR δ 3.92 (s, 3H), 3.95 (s, 3H), 3.97 (s, 3H), 6.21 (bs, 1H), 6.88 (d, *J* = 8.0 Hz, Ar, 1H), 6.98 (d, *J* = 8.4 Hz, Ar, 1H), 7.15 (d, *J* = 2 Hz, Ar, 1H), 7.28 (dd, *J* = 1.6, 8 Hz, Ar, 1H), 7.41 (d, *J* = 15.6 Hz, 1H), 7.62-7.70 (series of m, Ar, 2H), 7.75 (d, *J* = 15.6 Hz, 1H); ^{13}C NMR δ 55.97, 55.99, 56.12, 110.09, 110.48, 111.09, 113.71, 119.54, 122.95, 123.52, 128.03, 131.22, 144.15, 146.88, 149.21, 150.23, 151.25, 188.58; Anal. Calcd. for $C_{18}H_{18}O_5$: C, 68.78; H, 5.77; Found: C, 68.69; H, 5.56.

(E)-4-(4-hydroxy-3-methoxyphenyl)-1-((2S,3R,4R,5S,6R)-3,4,5-trihydroxy-6-(hydroxymethyl)tetrahydro-2H-pyran-2-yl)but-3-en-2-one **13**.

Compound **23** (0.30 g, 0.57 mmol) was stirred in sodium methoxide/methanol solution (0.006 g, 0.11 mmol in 10 mL) for 10 min. The reaction mixture was neutralized using a Dowex Marathon C (H+ form) resin, filtrated and concentrated *in vacuo* to obtain **13** as a brown solid. (0.19 g, 94%): mp = 54-55°C; $[\alpha]_D^{20}$ 11.7 (c 0.5, MeOH); ^{1}H NMR (CD_3OD) δ 2.86 (dd, *J* = 9.2, 16.0 Hz, 1H), 3.10 (dd, *J* = 2.8, 16.0 Hz, 1H), 3.14-3.41 (series of m, 4H), 3.62 (dd, *J* = 5.2, 12.4 Hz, 1H), 3.72-3.79 (series of m, 2H), 3.88 (s, 3H), 6.73 (d, *J* = 16.4 Hz, 1H), 6.81 (d, *J* = 8.0 Hz, Ar, 1H), 7.12 (dd, *J* = 2.0, 8.4 Hz, Ar, 1H), 7.21 (d, *J* = 2.0 Hz, Ar, 1H), 7.59 (d, *J* = 16.4 Hz, 1H); ^{13}C NMR δ (CD_3OD) 42.80, 55.06, 61.35, 70.25, 73.76, 76.16, 78.31, 80.16, 110.59, 115.16, 123.11, 123.34, 126.42, 144.51, 147.98, 149.54, 199.88; Anal. Calcd. for $C_{17}H_{22}O_8$: C, 57.62; H, 6.26; Found: C, 57.70; H, 6.31.

(2E,2′E)-1,1′-(5,5′,6,6′-tetramethoxy-[1,1′-biphenyl]-3,3′-diyl)bis(3-(4-hydroxy-3-methoxyphenyl)prop-2-en-1-one) **14**.

To a stirred solution of KOH (6.20 g, 110.51 mmol) in water (6 mL) cooled to 0 °C in an ice bath was added dropwise a solution of tetrahydropyranyl vanillin acetal [6] (1.16 g, 4.91 mmol) and *O*Me-dehydrodiapocynin [7] (0.44 g, 1.22 mmol) in methanol (30 mL). The reaction mixture was kept at 0 °C for 3 h, and then at room temperature for 12 h, under N_2. *p*-Toluenesulfonic acid (0.02 g, 0.12 mmol) was added and the solution was stirred for 2 h. The mixture was poured into ice–water (10 mL), adjusted to pH 3–4 with hydrochloridric acid (10% solution), and then extracted with ethyl acetate. The organic layer was successively washed with water and saturated brine, dried over anhydrous sodium sulphate and purified by flash chromatography using a 1:1 mixture of ethyl acetate:petroleum as eluent to give **14** as a yellow solid (0.61 g, 80%): mp = 209-210°C; ^{1}H NMR δ 3.77 (s, 6H), 3.92 (s, 6H), 4.01 (s, 6H), 6.01 (bs, 2H), 6.92 (d, *J* = 8.0 Hz, Ar, 2H), 7.11 (d, *J* = 2.0 Hz, Ar, 2H), 7.19 (dd, *J* = 2.0, 8.4 Hz, Ar, 2H), 7.35 (d, *J* = 16.0 Hz, 2H) 7.61 (d, *J* = 2.4 Hz, Ar, 2H), 7.71 (d, *J* = 2.4 Hz, Ar, 2H), 7.79 (d, *J* = 16.0 Hz, 2H); ^{13}C NMR δ 56.08, 56.09, 60.91, 110.07, 111.67, 114.83, 118.96, 123.51, 124.21, 127.43, 131.47, 133.88, 145.17, 146.78, 148.31, 150.87, 153.03, 188.87; Anal. Calcd. for $C_{36}H_{34}O_{10}$: C, 69.00; H, 5.47; Found: C, 69.18; H, 5.36.

General procedure for the synthesis of chalcones **15** *and* **16**

To a stirred solution of KOH (90 eq) in water (4 mL) cooled to 0 °C in an ice bath was added dropwise a solution of *O*Me-dehydidrovanillin [8] (1 eq) and 4-hydroxy-3-methoxyacetophenone (for **15**) or 2-hydroxy-5-methoxyacetophenone (for **16**) (3 eq) in methanol (20 mL). The reaction mixture was kept at 0 °C for 3 h, and then at room temperature for 96 h, under N_2. The mixture was poured into ice–water (10 mL), adjusted to pH 3–4 with hydrochloridric acid (10% solution), and then extracted with ethyl acetate. The organic layer was successively washed with water and saturated brine, dried over anhydrous sodium sulphate and purified by flash chromatography using a 2:1 mixture of ethyl acetate:petroleum as eluent to give **15** or **16**.

(2E,2′E)-3,3′-(5,5′,6,6′-tetramethoxy-[1,1′-biphenyl]-3,3′-diyl)bis(1-(4-hydroxy-3-methoxyphenyl)prop-2-en-1-one) **15**: yellow solid (70%): mp = 220-222°C; ^{1}H NMR δ 3.73 (s, 6H), 3.98 (s, 6H), 3.99 (s, 6H), 6.15 (bs, 2H), 6.98 (d, *J* = 8.0 Hz, Ar, 2H), 7.19 (d, *J* = 2.0 Hz, Ar, 2H), 7.20 (d, *J* = 2.0 Hz, Ar, 2H), 7.47 (d, *J* = 15.6 Hz, 2H), 7.64 (m, Ar, 4H), 7.76 (d, *J* = 15.6 Hz, 2H); ^{13}C NMR δ 56.01, 56.16, 60.91, 110.42, 111.83, 113.73, 120.93, 123.45,

123.68, 130.55, 131.04, 132.48, 143.66, 146.88, 148.91, 150.33, 152.96, 188.39; Anal. Calcd. for $C_{36}H_{34}O_{10}$: C, 69.00; H, 5.47; Found: C, 69.09; H, 5.46.

2E,2′E)-3,3′-(5,5′,6,6′-tetramethoxy-[1,1′-biphenyl]-3,3′-diyl)bis(1-(2-hydroxy-5-methoxyphenyl)prop-2-en-1-one) **16**: yellow solid (55%): mp = 190-192°C; ^{1}H NMR δ 3.75 (s, 6H), 3.82 (s, 6H), 3.99 (s, 6H), 6.96 (d, *J* = 8.8 Hz, Ar, 2H), 7.13 (dd, *J* = 2.8, 8.8 Hz, Ar, 2H), 7.21 (d, *J* = 2.0 Hz, Ar, 2H), 7.24 (d, *J* = 2.0 Hz, Ar, 2H), 7.35 (d, *J* = 2.8 Hz, Ar, 2H), 7.51 (d, *J* = 16.0 Hz, 2H), 7.89 (d, *J* = 16.0 Hz, 2H); ^{13}C NMR δ 56.06, 56.23, 60.95, 112.18, 113.42, 119.25, 119.32, 119.70, 123.40, 123.84, 130.06, 132.48, 145.33, 149.46, 151.65, 153.02, 157.84, 193.18; Anal. Calcd. for $C_{36}H_{34}O_{10}$: C, 69.00; H, 5.47; Found: C, 69.15; H, 5.41.

(2E,2′E)-diethyl 3,3′-(6,6′-dihydroxy-5,5′-dimethoxy-[1,1′-biphenyl]-3,3′-diyl)diacrylate **17**.

To a stirred solution of ferulic acid dimer [9] (1.11 g, 2.87 mmol) in absolute ethanol (10 mL) was added concentrated sulphuric acid (0.1 mL) The reaction mixture was stirred at reflux for 12 h. Water (100 mL) and dichloromethane (100 mL) were added. The organic layer was successively washed with an aqueous saturated solution of sodium bicarbonate, dried over anhydrous sodium sulphate and evaporated to obtain pure **17** as a brown solid. (1.20 g, 95%): mp = 100-102°C; ^{1}H NMR δ 1.33 (t, *J* = 7.2 Hz, 6H), 3.97 (s, 6H), 4.24 (q, *J* = 7.2 Hz, 4H), 6.21 (s, 2H), 6.32 (d, *J* = 15.6 Hz, 2H), 7.07 (d, *J* = 2.0 Hz, Ar, 2H), 7.14 (d, *J* = 2.0 Hz, Ar, 2H), 7.62 (d, *J* = 15.6 Hz, 2H); ^{13}C NMR δ 14.34, 56.18, 60.38, 108.72, 116.19, 123.55, 124.82, 126.73, 144.49, 145.07, 147.25, 167.18; Anal. Calcd. for $C_{24}H_{26}O_8$: C, 65.15; H, 5.92; Found: C, 65.11; H, 5.91.

(2R,3R,4R,5S,6S)-2-(acetoxymethyl)-6-((E)-4-(4-hydroxy-3-methoxyphenyl)-2-oxobut-3-en-1-yl)tetrahydro-2H-pyran-3,4,5-triyl triacetate **23**.

To a stirred solution of tetrahydropyranyl vanillin acetal [6] (0.52 g, 2.22 mmol) and *per-O*-acetylated-β-C-glucopyranosyl ketone [10] (0.86 g, 2.22 mmol) in dichloromethane (30 ml) was added pyrrolidine (0.3 mL). The reaction mixture was stirred for 72 h, then was poured into ice–water (10 ml), adjusted to pH 3–4 with hydrochloridric acid (10% solution). The organic layer was successively washed with water and saturated brine, dried over anhydrous sodium sulphate and evaporated. Methanol (30 mL) and pyridinium-*p*-toluenesulfonate (0.05g, 0.21 mmol) were added and the solution was stirred for 3 h. The mixture was evaporated to give a yellow oil that was purified by flash chromatography using a 2:5 mixture of petroleum: acetone as eluent to obtain **23** as a yellow solid (0.95 g, 82%): mp = 59-60°C; $[\alpha]_D^{20}$ -10.0 (c = 0.5, $CHCl_3$); ^{1}H NMR δ 2.00 (s, 3H), 2.01 (s, 3H), 2.02 (s, 3H), 2.03 (s, 3H), 2.65 (dd, *J* = 3.2, 16 Hz, 1H), 2.99 (dd, *J* = 8.4, 16 Hz, 1H), 3.71 (dt, *J* = 2.0, 12.8 Hz, 1H), 3.94 (s, 3H), 4.03 (dd, *J* = 1.6, 12.4 Hz, 1H), 4.12 (m, 1H), 4.26 (dd, *J* = 4.8, 12.4 Hz, 1H), 4.98 (t, *J* = 9.6 Hz, 1H), 5.07 (t, *J* = 9.2 Hz, 1H), 5.22 (t, *J* = 9.6 Hz, 1H), 5.93 (bs, 1H), 6.63 (d, *J* = 16.4 Hz, 1H), 6.93 (d, *J* = 8.0 Hz, Ar, 1H), 7.05 (d, *J* = 1.6 Hz, Ar, 1H), 7.11 (dd, *J* = 1.6, 8.0 Hz, Ar, 1H), 7.49 (d, *J* = 16.4 Hz, 1H); ^{13}C NMR δ 20.62, 20.64, 20.67, 20.74, 42.39, 55.97, 62.03, 68.51, 71.71, 74.19, 74.22, 75.71, 109.47, 114.85, 123.76, 124.01, 127.72, 144.02, 146.84, 148.48, 169.58, 170.03, 170.24, 170.65, 196.01; Anal. Calcd. for $C_{25}H_{30}O_{12}$: C, 57.47; H, 5.79; Found: C, 57.38; H, 5.76.

1.2 Spectrophotometric assay

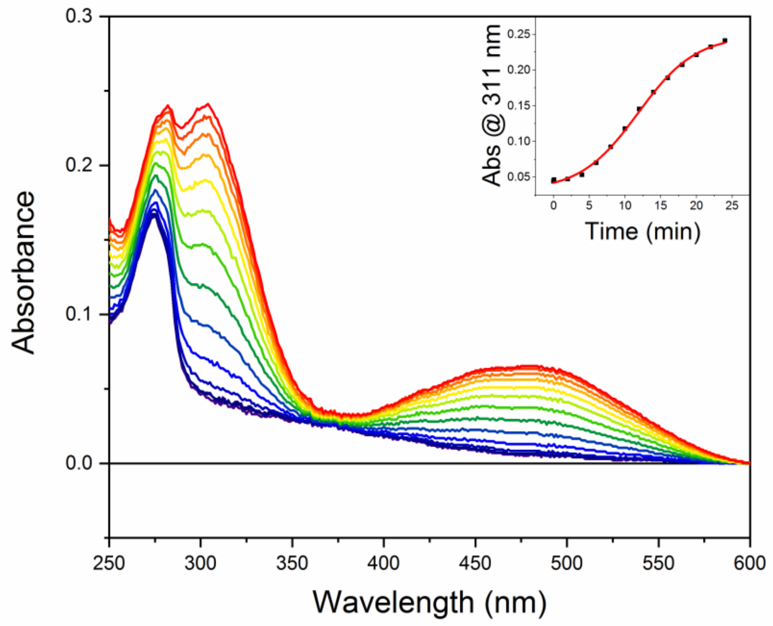

Figure S1. UV-Vis spectra of tyrosine (90 μM) in 50 mM phosphate buffer, pH 6.8, at different time after the addition of tyrosinase (83 U/mL). In the insert, the time-course of tyrosine oxidation detected at 311 nm.

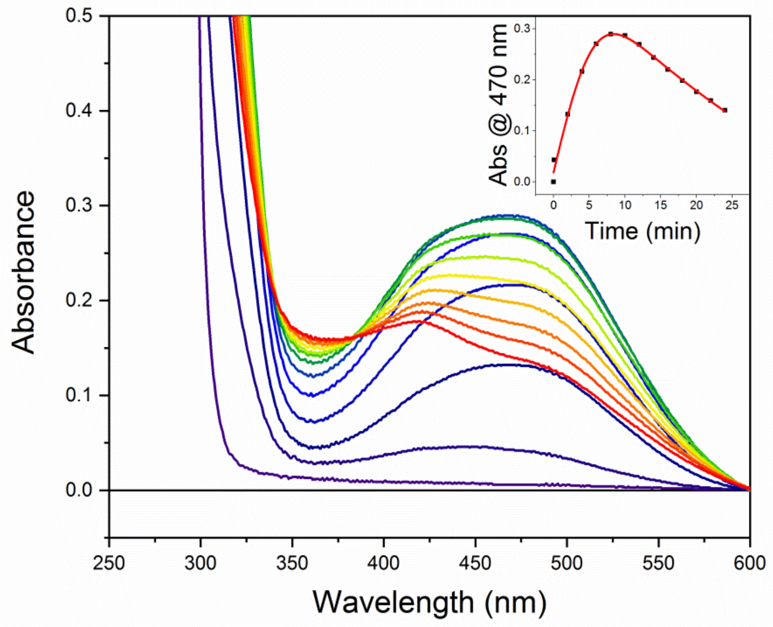

Figure S2. UV-Vis spectra of dopamine (9 μM) in 50 mM phosphate buffer, pH 6.8, at different time after the addition of tyrosinase (8.3 U/mL). In the insert, the time-course of dopamine oxidation detected at 472 nm.

1.3 Electrochemical assays: biosensor calibration and inhibition protocols

Preparation of Tyrosinase and Laccase biosensors

The biosensors were prepared starting from a Teflon™-insulated silver wire (30 mm in length; Ø = 125 μm), as previously described [11, 12]. Briefly, 1 mm of the silver wire was exposed and then introduced into a silica capillary cylinder (10 mm in length; I.D. Ø = 180 μm, Polymicro Technologies, Phoenix, AZ, USA) partly packed with graphite-loaded (55% w/w) epoxy resin. By mixing 850 mg of graphite with 500 mg of Araldite-M and 200 mg of hardener, a preliminary disc electrode of carbon-composite was fabricated (Ø = 180 μm; area: 2.5×10^{-4} cm^2) by filling the silica capillary tubing with the mixture. The silver wire assured a good electrical contact. After 24 h at 40 °C, the electrode surface was cleaned using a high-speed drill (Dremel® 300) equipped with an aluminum oxide grinding wheel. On top of the electrode surface a 1 μl of Tyrosinase or Laccase solution was deposited. After the complete evaporation of water at room temperature, the biosensor was quickly dipped in the polyurethane solution in order to entrap the enzyme on the electrode surface, and after the complete evaporation of the solvent biosensors were put to stabilize in PBS pH=6.0 overnight.

Biosensor Calibration protocol

The electrochemical measurements were performed at room temperature in a classical three-electrode electrochemical cell consisting of four biosensors as working electrodes, a reference electrode (Ag/AgCl in NaCl, 3.0 M) and the auxiliary electrode (a large surface steel wire), as previously described [13, 14].
In order to characterize tyrosinase and laccase biosensors' performances, a preliminary cyclic voltammetry was performed in PBS pH=6.0, using dopamine as reference compound, so that to fix the working reduction potential of the corresponding quinone, obtained by the oxidation of the amine. In parallel, cyclic voltammetries of inhibitors have been carried out in the same conditions as dopamine to determine any eventual overlapping of peaks in the reduction region and evaluate any possible interfering current. The voltammograms (data not shown) were obtained in a potential range from −0.5 V to +0.5 V at 100 mV s^{-1} of scan rate.

Constant potential amperometry (CPA) was used for in-vitro calibrations and for inhibition evaluation experiments by applying a constant potential of -50 mV against an Ag/AgCl reference electrode, by means of a four-channel potentiostat (eDAQ Quadstat, e- Corder 410, eDAQ Europe, Poland) and the software Chart (v 5.5, eDAQ Europe, Poland). Thus, once the stabilization of the baseline of biosensors was reached, increasing volumes of a stock solution of dopamine 1 M were added in order to obtain different concentrations ranging from 0 to 140 mM. Biosensors were then characterized in terms of Michaelis–Menten kinetics (V_{MAX} and apparent K_M) as parameters taken into account to determine the possible inhibition effects of the studied molecules. Statistical differences of parameters were evaluated by means of t-test ($p<0.05$). The statistical software GraphPad Prism v 5.02 was used for the evaluation of enzymatic kinetic parameters and IC_{50} values and to perform t-test.

Biosensor Inhibition protocols

Two different inhibition protocols have been used. The first has been set in order to assess the IC50 values of different inhibitors, as previously demonstrated. [15, 16]. Briefly, a fixed concentration of dopamine (50 μM in 10 ml of PBS pH=6.0 at a fixed potential of -50 mV vs Ag/AgCl) has been injected in the electrochemical cell. After having reached a stable baseline, known volumes of a stock solution of each inhibitor (10 mM) have been added in the cell up to the desired concentration. The second protocol has been explained in the manuscript

at 4.3. paragraph of the Materials and Methods section. For each inhibitor was built a group of biosensors (n=4) for both tyrosinase and laccase enzyme.

1.4 Viability and oxidative stress assays

PC12 Cells Culture

PC12 cells, rat pheochromocytoma-derived cell line (ATCCCRL-1721) (passages 12–25) were maintained at 37 °C humidified air containing 5% CO_2/95% atmospheric air and cultured in a 60 mm plastic culture plates with Dulbecco's modified Eagle's medium supplemented with 10% horse serum, 5% fetal bovine serum and 1% of penicillin/streptomycin. PC12 cells were treated for 24 h with the different compounds at different concentrations ranging from 1 to 40 μM, to evaluate their eventual toxicity. Then, in order to assess the eventual compounds' protection properties, PC12 cell treated with hydrogen peroxide (100μM) in association with increasing concentration of the above-mentioned molecules, from 1 up to 20 μM. For the compounds that had been shown to protect cells from the oxidative insult from H_2O_2, a co-treatment with $MnCl_2$ 1 mM and the compounds was performed, in order to evaluate whether the same compound could also able to protect against $MnCl_2$ insult. For all treatments cell viability was measured by means of MTT assay, as explained in the following paragraph [17].

MTT Assay

At the end of the exposure time of each experiment, the cell viability was assessed by means of MTT (3-(4,5-dimethyl-thiazol-2-yl)-2,5-diphenyltetrazoliumbromide) assay. For this purpose, 1 mg/mL of MTT was added to each sample and incubated for 4 h at 37 °C. Only viable cells are able to convert the soluble dye MTT into the insoluble formazan crystals. After the incubation the MTT supernatant solution was removed, the cells were washed in phosphate-buffered saline (PBS) and centrifuged while the pellet was dissolved in 2 mL of isopropanol. After centrifugation of the solution at 4,000 rpm for 5 min, the absorbance for each sample was assessed by means of a Bauty Diagnostic Microplate Reader at 578 nm. All experiments were performed in 24-well plates (1×10^5 cells/mL/well) and repeated in triplicate.

Table S1. Kinetic parameters (V_{MAX} and K_M values) obtained from biosensors' calibrations, without and with the inhibitor. For each inhibitor was built a group of biosensors (n=4) for both tyrosinase (TYR) and laccase (LAC) enzyme and parameters' variations were reported. Statistical significance (p<0.05) was evaluate by means of t-test.

INHIBITOR	ENZYME	SUBSTRATE (DA)		SUBSTRATE + INHIBITOR	
		V_{MAX} (nA)	K_M (mM)	V_{MAX} (nA)	K_M (mM)
**7**	**TYR**	127.2±9.9	0.430±0.181	114.1±12.5	20.030±7.262*
	**LAC**	218.9±7.1	2.292±0.363	188.4±9.0	17.840±2.907*
**8**	**TYR**	47.6±2.2	1.370±0.322	35.2±1.0*	5.769±0.719*
	**LAC**	99.3±11.3	6.156±1.006	78.7±8.8	9.292±1.677*
**9**	**TYR**	224.4±21.6	1.646±0.612	183.2±11.1	9.158±1.762*
	**LAC**	119.9±9.6	8.658±2.728	90.5±7.4	14.340±4.170*
**10**	**TYR**	59.6±3.2	1.334±0.366	31.5±2.9*	7.136±2.729*
	**LAC**	107.7±12.0	9.400±1.489	89.2±7.7	12.700±1.327*
**11**	**TYR**	49.9±1.6	0.596±0.079	33.0±1.1	8.757±1.178*
	**LAC**	107.7±6.7	7.716±1.932	91.0±9.2*	24.450±7.731*
**12**	**TYR**	80.9±3.2	9.330±1.900	63.0±1.962*	11.050±1.409
	**LAC**	65.1±1.9	4.338±0.741	49.5±6.1	9.640±0.549*
**13**	**TYR**	66.9 ±2.0	4.489±0.821	37.3 ±4.0*	9.950±1.284*
	**LAC**	147.6±21.9	4.727±0.634	114.4±9.5	10.450±1.423*
**14**	**TYR**	69.6±3.3	0.504±0.139	48.8±4.67*	7.886±3.033*
	**LAC**	133.5±3.3	3.667±0.369	110.9±2.4*	7.405±0.578*
**15**	**TYR**	83.4±3.5	0.975±0.180	54.3±2.6	9.172±1.714*
	**LAC**	103.8±3.5	4.439±0.582	93.8±2.9	9.177±0.564*
**16**	**TYR**	38.1±3.4	0.569±0.217	22.2±0.6	10.010±1.002*
	**LAC**	151.5±10.7	6.390±1.655	139.9±6.4	9.933±1.741*
**17**	**TYR**	122.4±7.2	1.334±0.366	73.7±26*	7.136±2.729*
	**LAC**	115.9±13.23	9.304±0.503	96.5±8.3	12.600±0.559*
**18**	**TYR**	194.5±14.9	2.851±0.708	145.8±17.8	11.990±3.294*
	**LAC**	120.1±7.2	5.894±1.505	98.1±3.3	12.380±1.512*
**19**	**TYR**	285.6±10.4	7.399±0.688	123.5±9.8*	9.148±1.599
	**LAC**	124.5±11.1	3.654±1.489	111.4±8.9	7.204±2.640*
**20**	**TYR**	87.9±6.0	6.700±0.770	77.4±4.3	11.500±1.142*
	**LAC**	166.2±12.5	4.182±1.415	151.6±12.1	8.822±2.776*

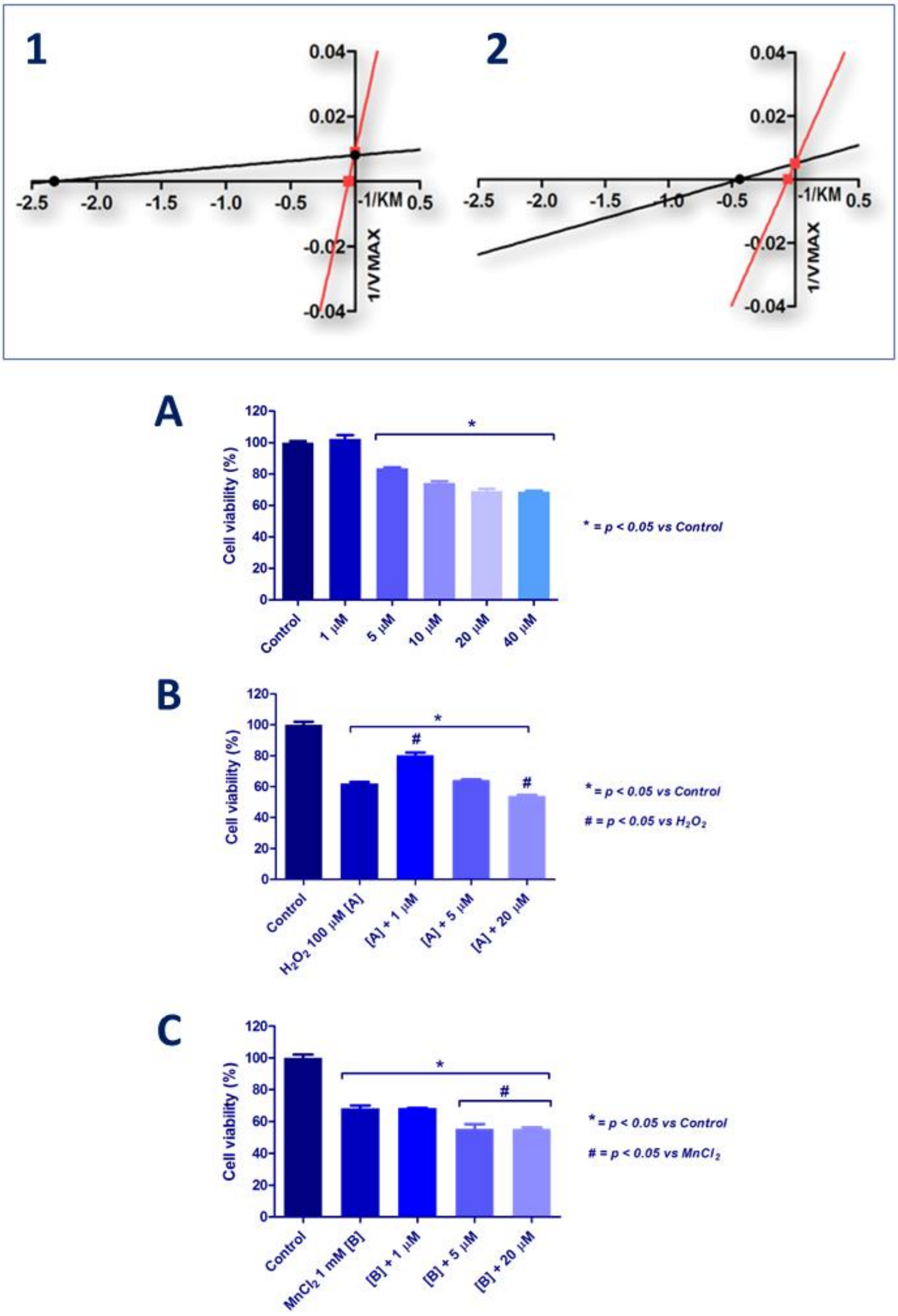

Figure S3: Lineweaver–Burk plot for compound **7** on tyrosinase (inset, 1) and laccase (inset, 2) enzymes. Black line: not inhibited enzyme; red line: inhibited enzyme. Panel A describes the effect of different concentrations of compound **7,** ranging from 1 up to 40 μM, on viability of PC12 cells; Panel B and C show the study of potential protective effects of compound **7** on damage induced by H_2O_2 (100 μM) or $MnCl_2$ (1 mM). MTT assay was performed on PC12 cells 24 h after each treatment. *$p<0.05$ vs control; #$p<0.05$ vs H_2O_2 or $MnCl_2$

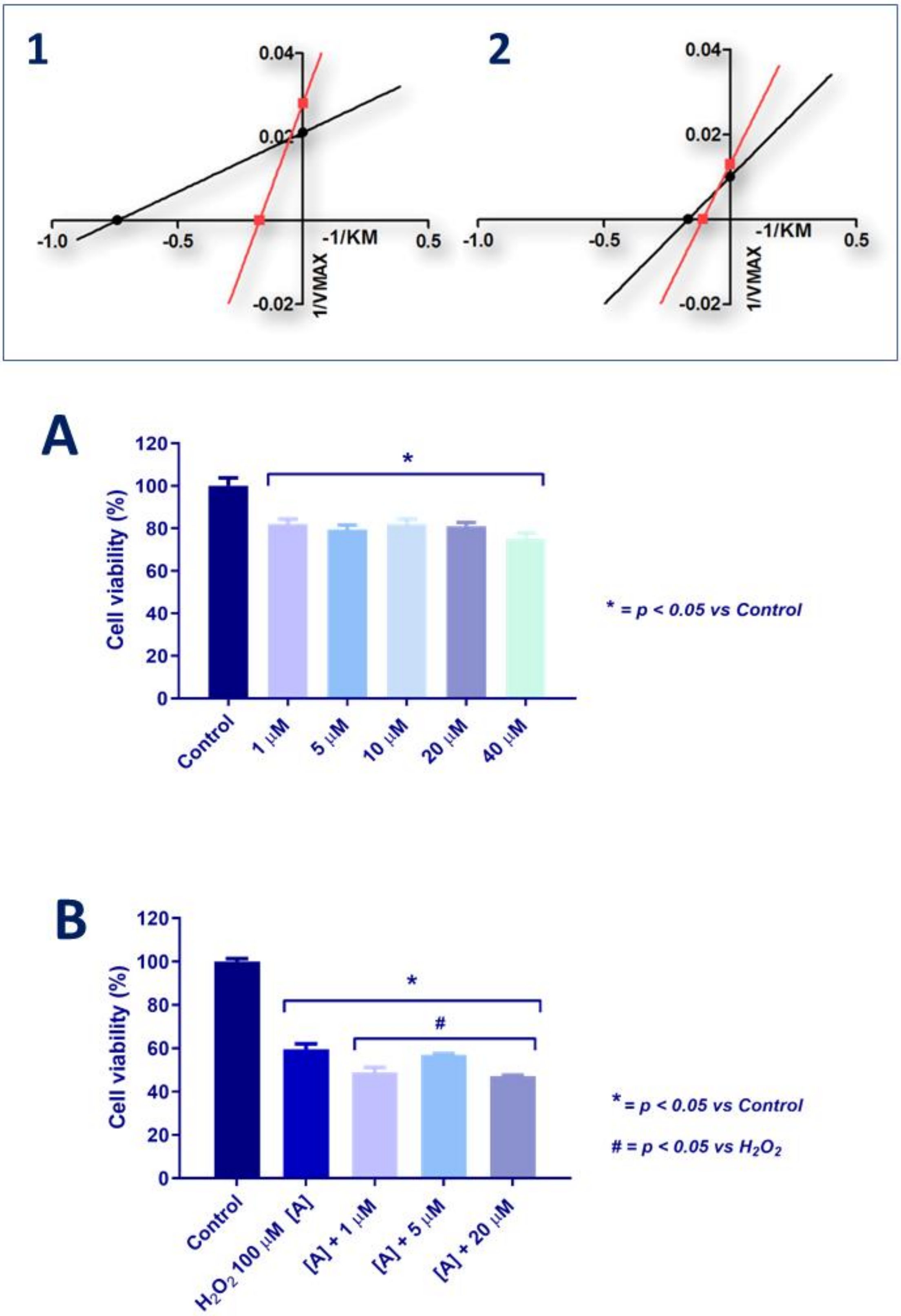

Figure S4: Lineweaver–Burk plot for compound **8** on tyrosinase (inset, 1) and laccase (inset, 2) enzymes. Black line: not inhibited enzyme; red line: inhibited enzyme. Panel A describes the effect of different concentrations of compound **8**, ranging from 1 up to 40 μM, on viability of PC12 cells; Panel B shows the study of potential protective effects of compound **8** on damage induced by H_2O_2 (100 μM). MTT assay was performed on PC12 cells 24 h after each treatment. *p<0.05 vs control; #p<0.05 vs H_2O_2

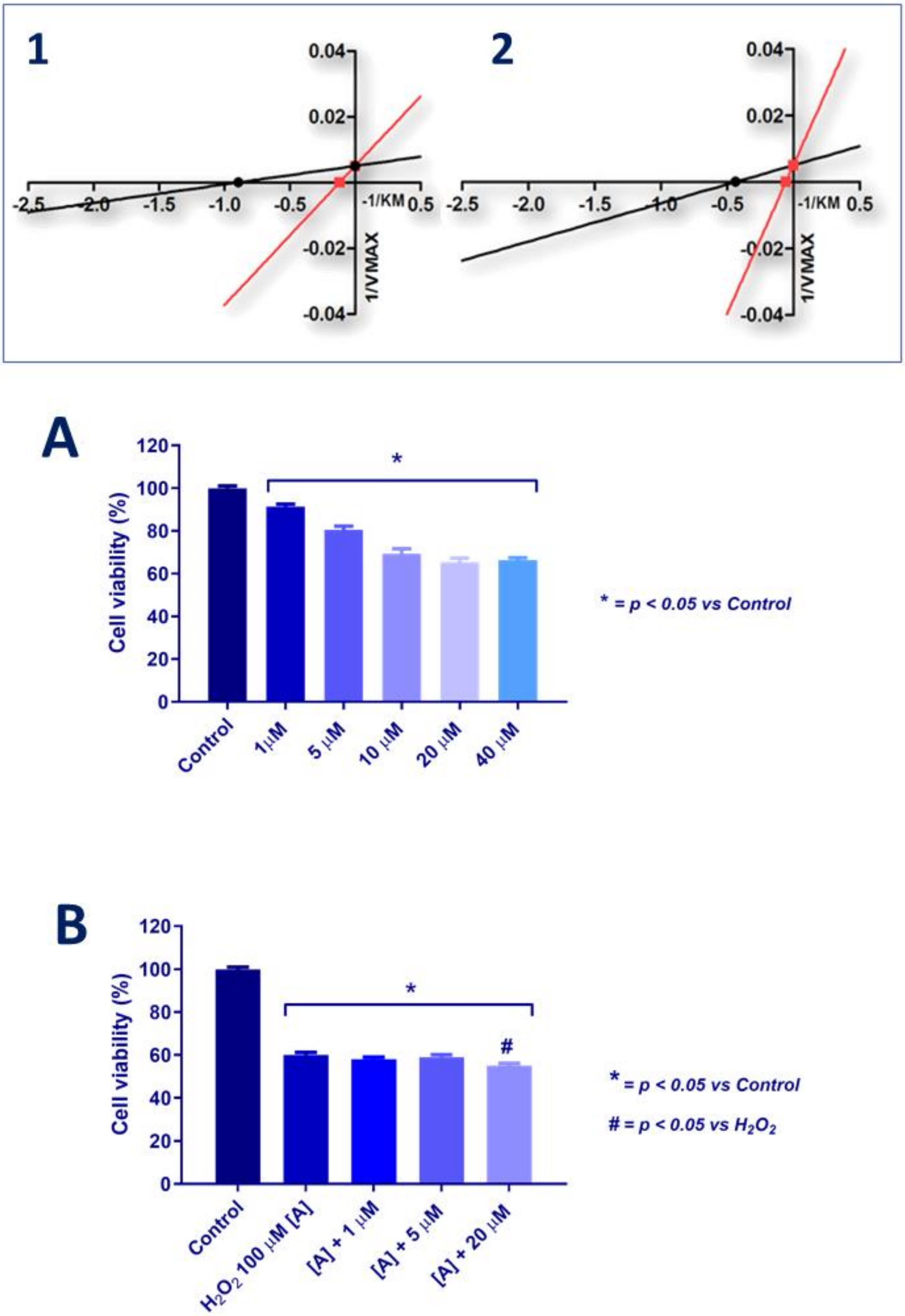

Figure S5: Lineweaver–Burk plot for compound **9** on tyrosinase (inset, 1) and laccase (inset, 2) enzymes. Black line: not inhibited enzyme; red line: inhibited enzyme. Panel A describes the effect of different concentrations of compound **9**, ranging from 1 up to 40 μM, on viability of PC12 cells; Panel B shows the study of potential protective effects of compound **9** on damage induced by H_2O_2 (100 μM). MTT assay was performed on PC12 cells 24 h after each treatment. *p<0.05 vs control; #p<0.05 vs H_2O_2.

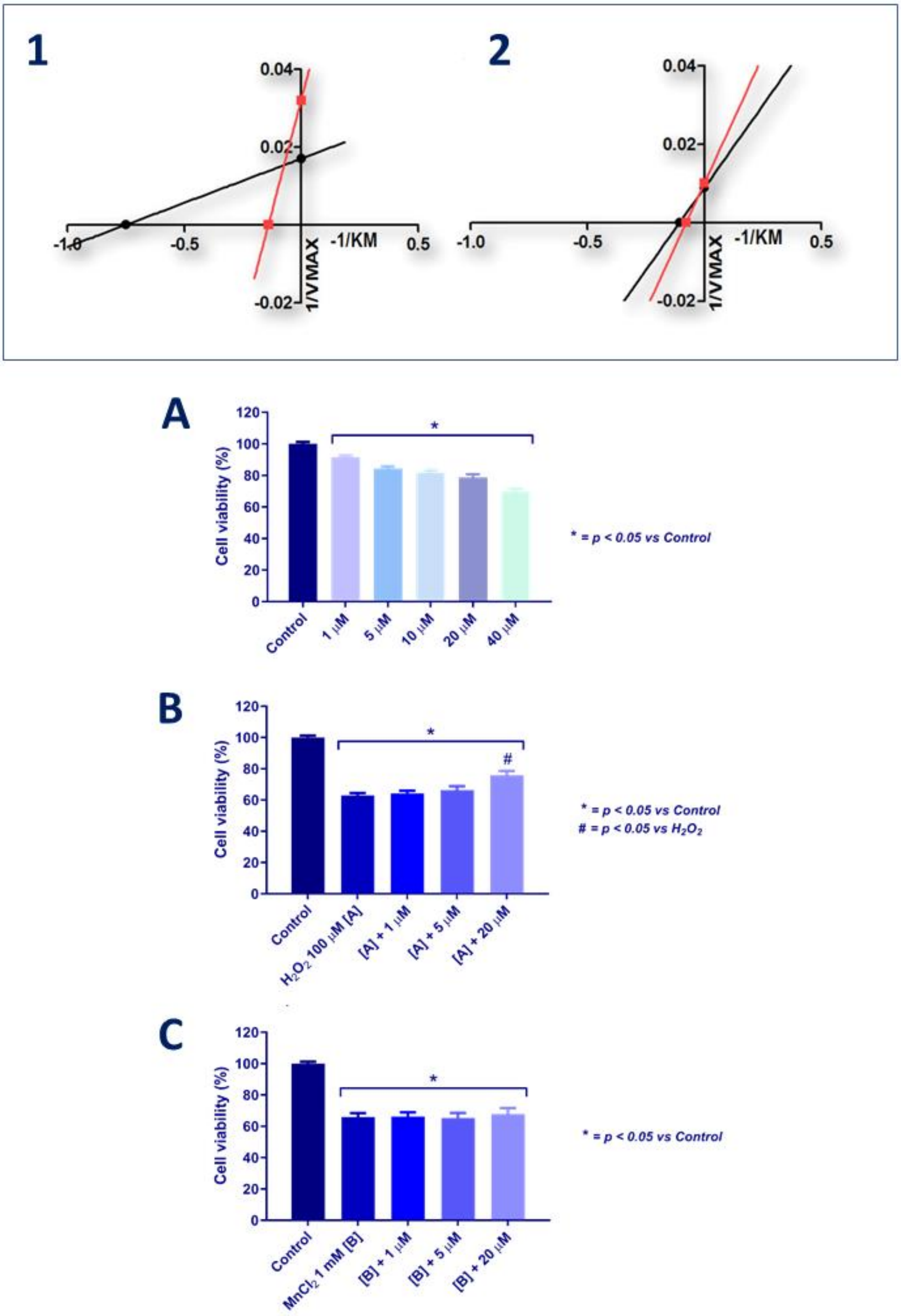

Figure S6: Lineweaver–Burk plot for compound **10** on tyrosinase (inset, 1) and laccase (inset, 2) enzymes. Black line: not inhibited enzyme; red line: inhibited enzyme. Panel A describes the effect of different concentrations of compound **10,** ranging from 1 up to 40 μM, on viability of PC12 cells; Panel B and C show the study of potential protective effects of compound **10** on damage induced by H_2O_2 (100 μM) or $MnCl_2$ (1 mM). MTT assay was performed on PC12 cells 24 h after each treatment. *p<0.05 vs control; #p<0.05 vs H_2O_2 or $MnCl_2$

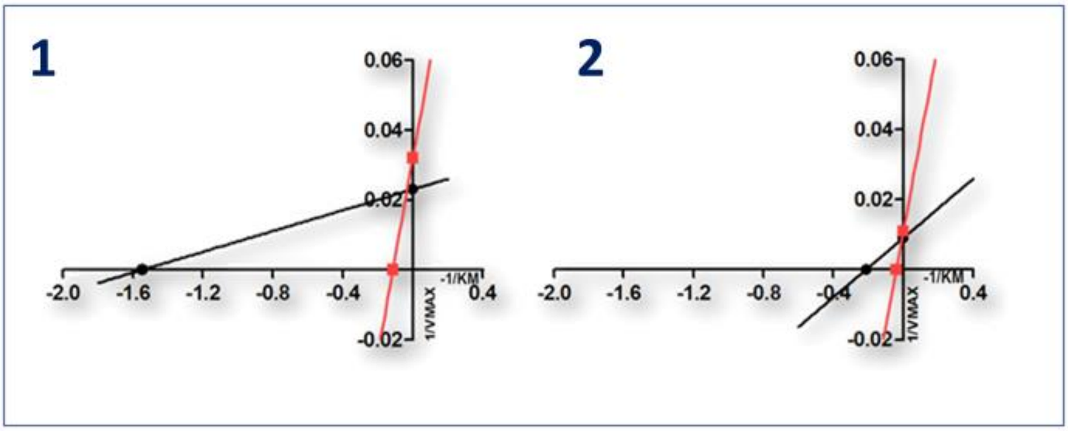

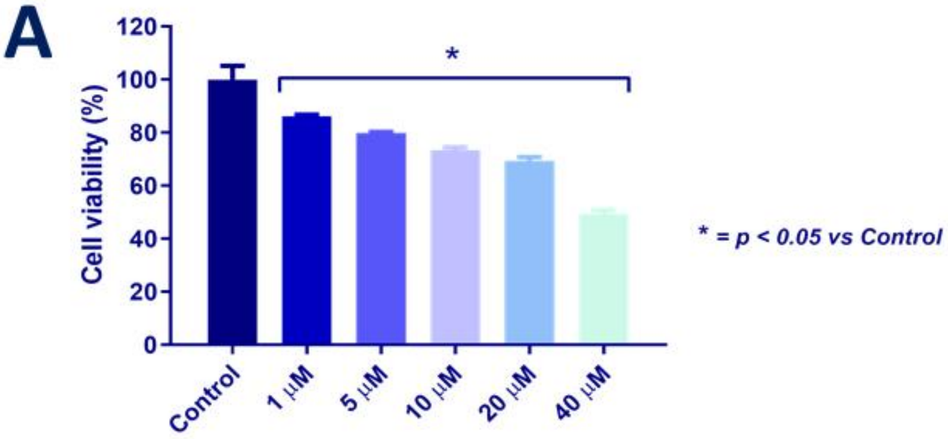

Figure S7: Lineweaver–Burk plot for compound **11** on tyrosinase (inset, 1) and laccase (inset, 2) enzymes. Black line: not inhibited enzyme; red line: inhibited enzyme.Panel A describes the effect of different concentrations of compound **11,** ranging from 1 up to 40 μM, on viability of PC12 cells. MTT assay was performed on PC12 cells 24 h after each treatment. *$p<0.05$ vs control.

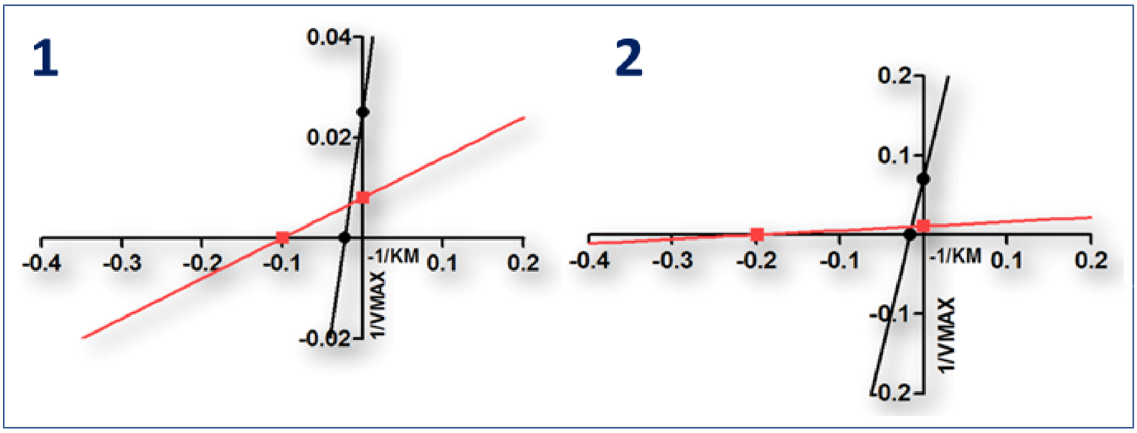

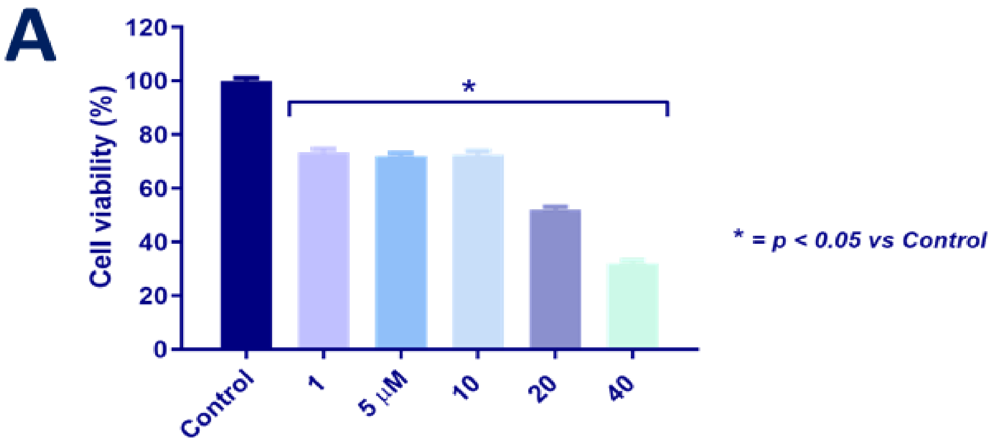

Figure S8: Lineweaver–Burk plot for compound **12** on tyrosinase (inset, 1) and laccase (inset, 2) enzymes. Black line: not inhibited enzyme; red line: inhibited enzyme.Panel A describes the effect of different concentrations of compound **12,** ranging from 1 up to 40 μM, on viability of PC12 cells. MTT assay was performed on PC12 cells 24 h after each treatment. *p<0.05 vs control.

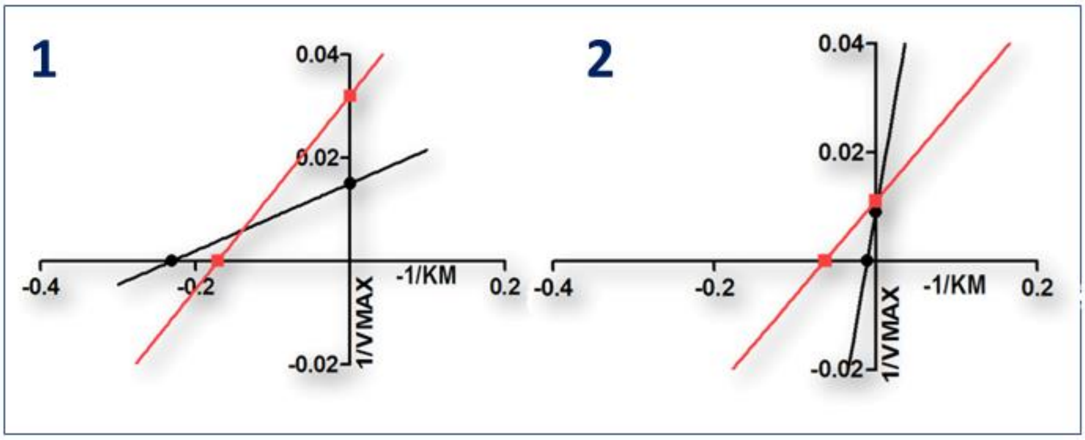

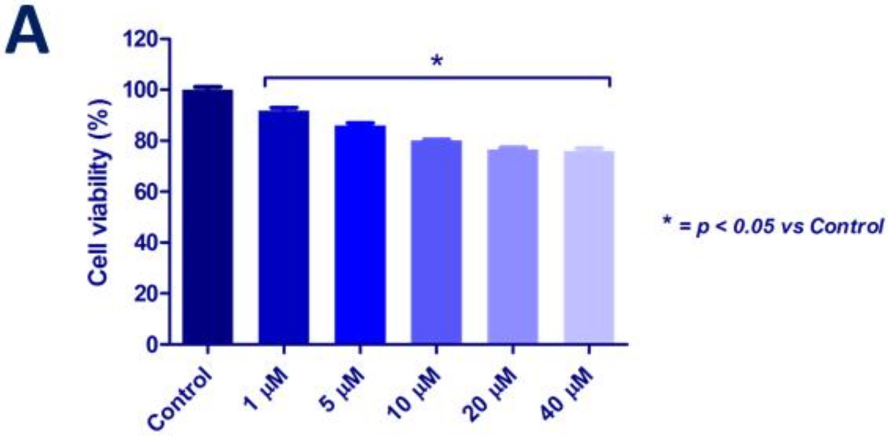

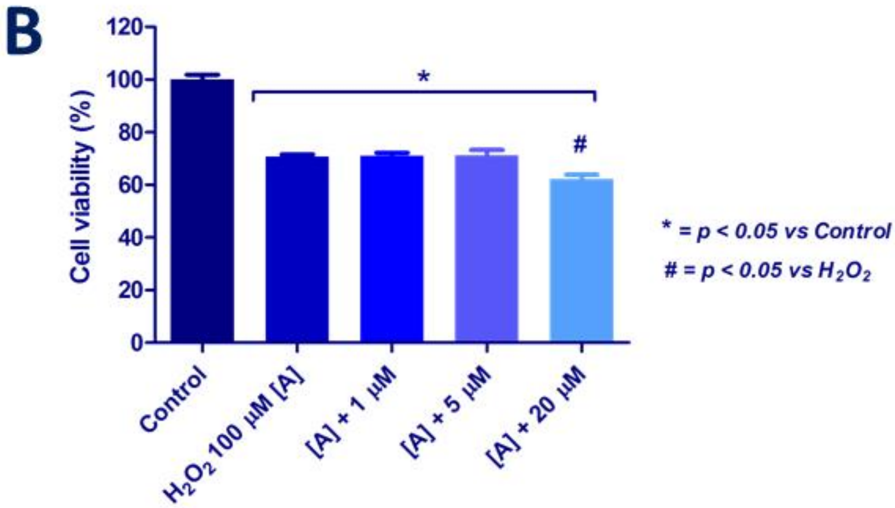

Figure S9: Lineweaver–Burk plot for compound **13** on tyrosinase (inset, 1) and laccase (inset, 2) enzymes. Black line: not inhibited enzyme; red line: inhibited enzyme. Panel A describes the effect of different concentrations of compound **13,** ranging from 1 up to 40 µM, on viability of PC12 cells; Panel B shows the study of potential protective effects of compound **13** on damage induced by H_2O_2 (100 µM). MTT assay was performed on PC12 cells 24 h after each treatment. *p<0.05 vs control; #p<0.05 vs H_2O_2.

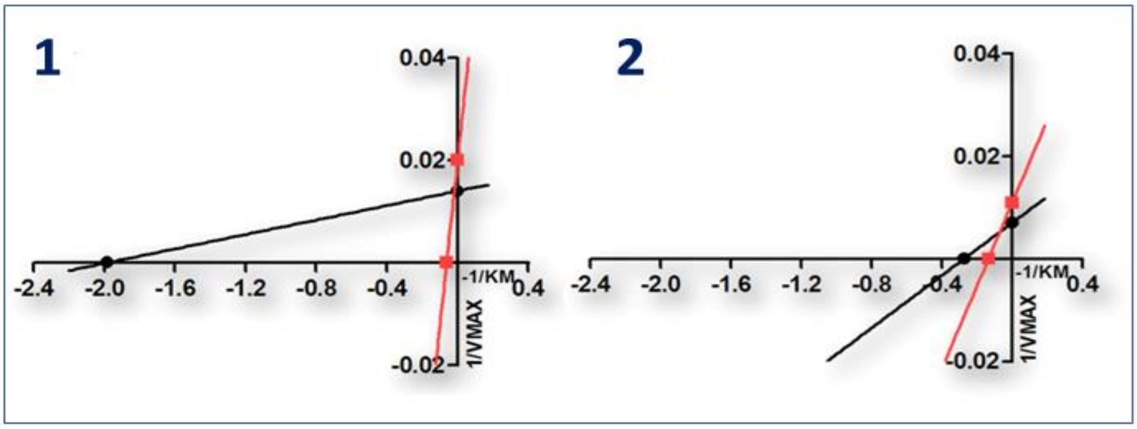

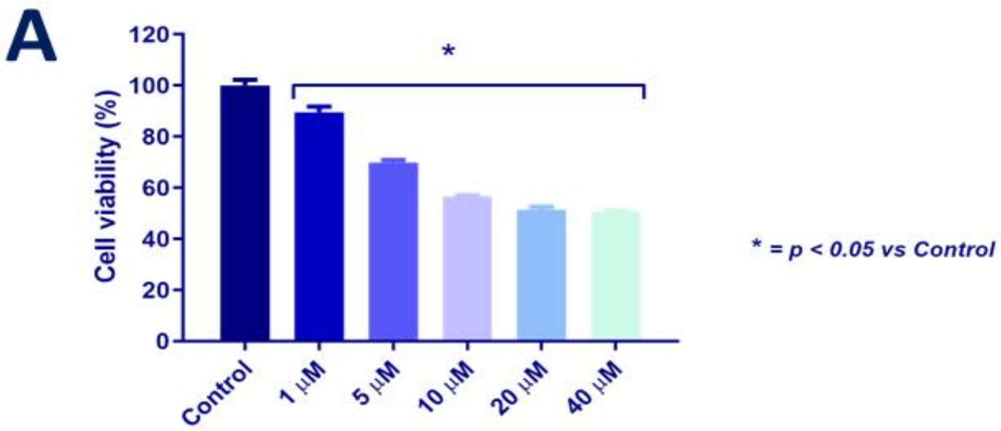

Figure S10: Lineweaver–Burk plot for compound **14** on tyrosinase (inset, 1) and laccase (inset, 2) enzymes. Black line: not inhibited enzyme; red line: inhibited enzyme. Panel A describes the effect of different concentrations of compound **14,** ranging from 1 up to 40 µM, on viability of PC12 cells. MTT assay was performed on PC12 cells 24 h after each treatment. *$p<0.05$ vs control.

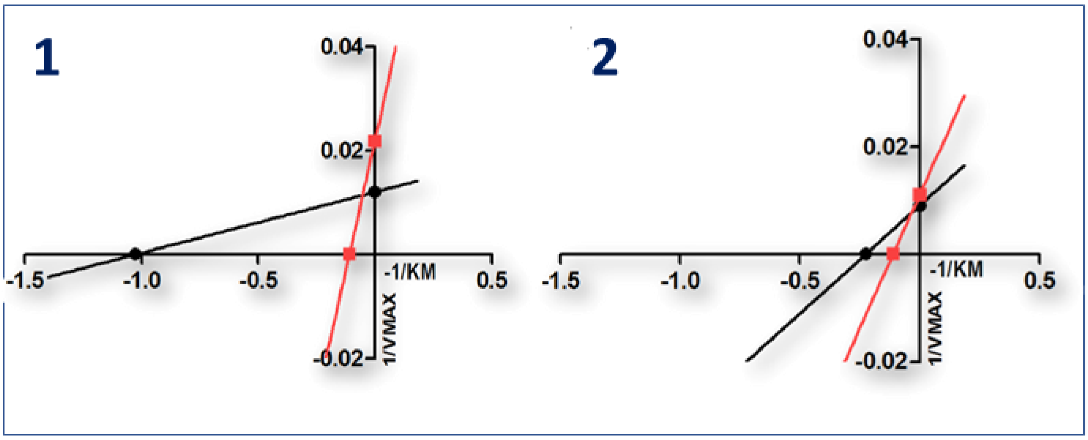

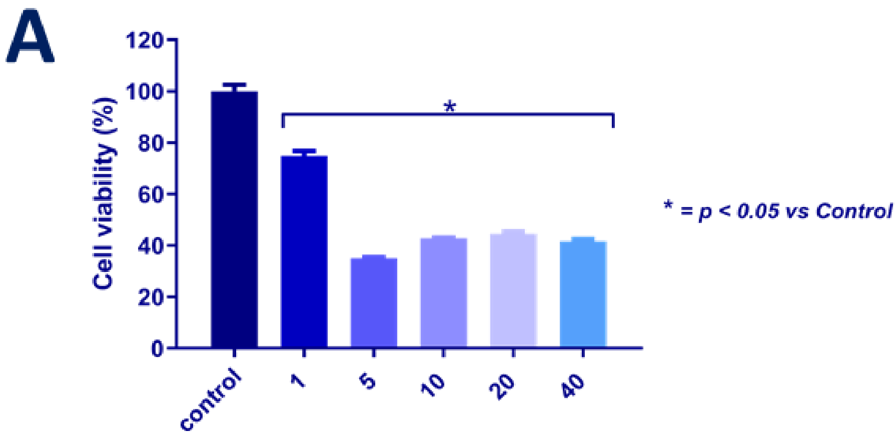

Figure S11: Lineweaver–Burk plot for compound **15** on tyrosinase (inset, 1) and laccase (inset, 2) enzymes. Black line: not inhibited enzyme; red line: inhibited enzyme. Panel A describes the effect of different concentrations of compound **15,** ranging from 1 up to 40 μM, on viability of PC12 cells. MTT assay was performed on PC12 cells 24 h after each treatment. *p<0.05 vs control.

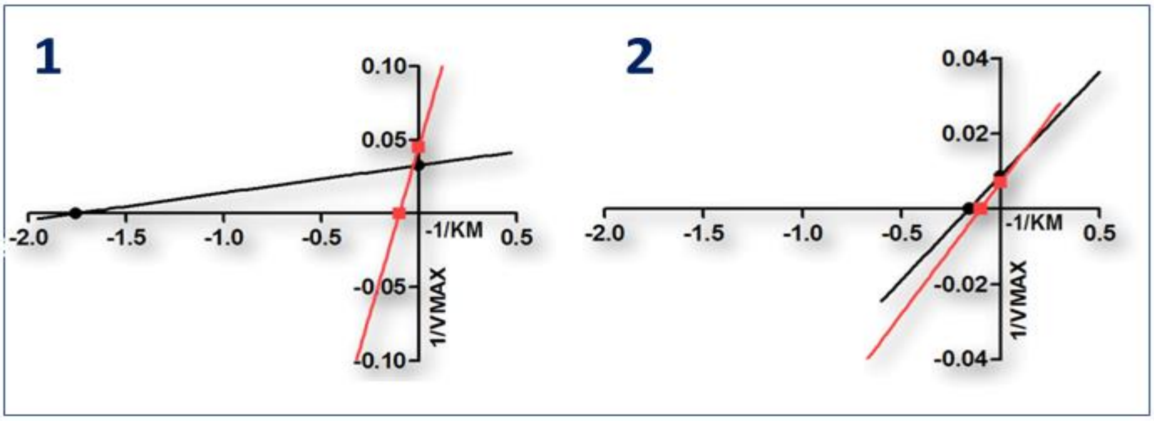

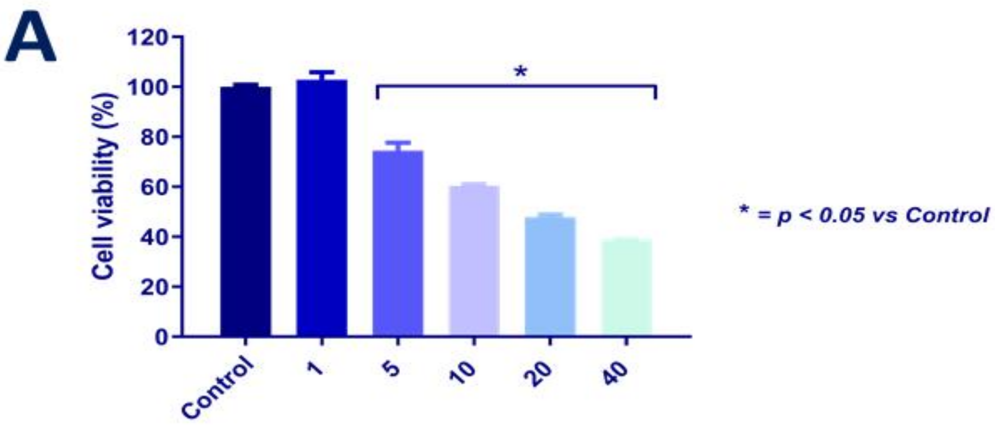

Figure S12: Lineweaver–Burk plot for compound **16** on tyrosinase (inset, 1) and laccase (inset, 2) enzymes. Black line: not inhibited enzyme; red line: inhibited enzyme.Panel A describes the effect of different concentrations of compound **16,** ranging from 1 up to 40 μM, on viability of PC12 cells. MTT assay was performed on PC12 cells 24 h after each treatment. *p<0.05 vs control.

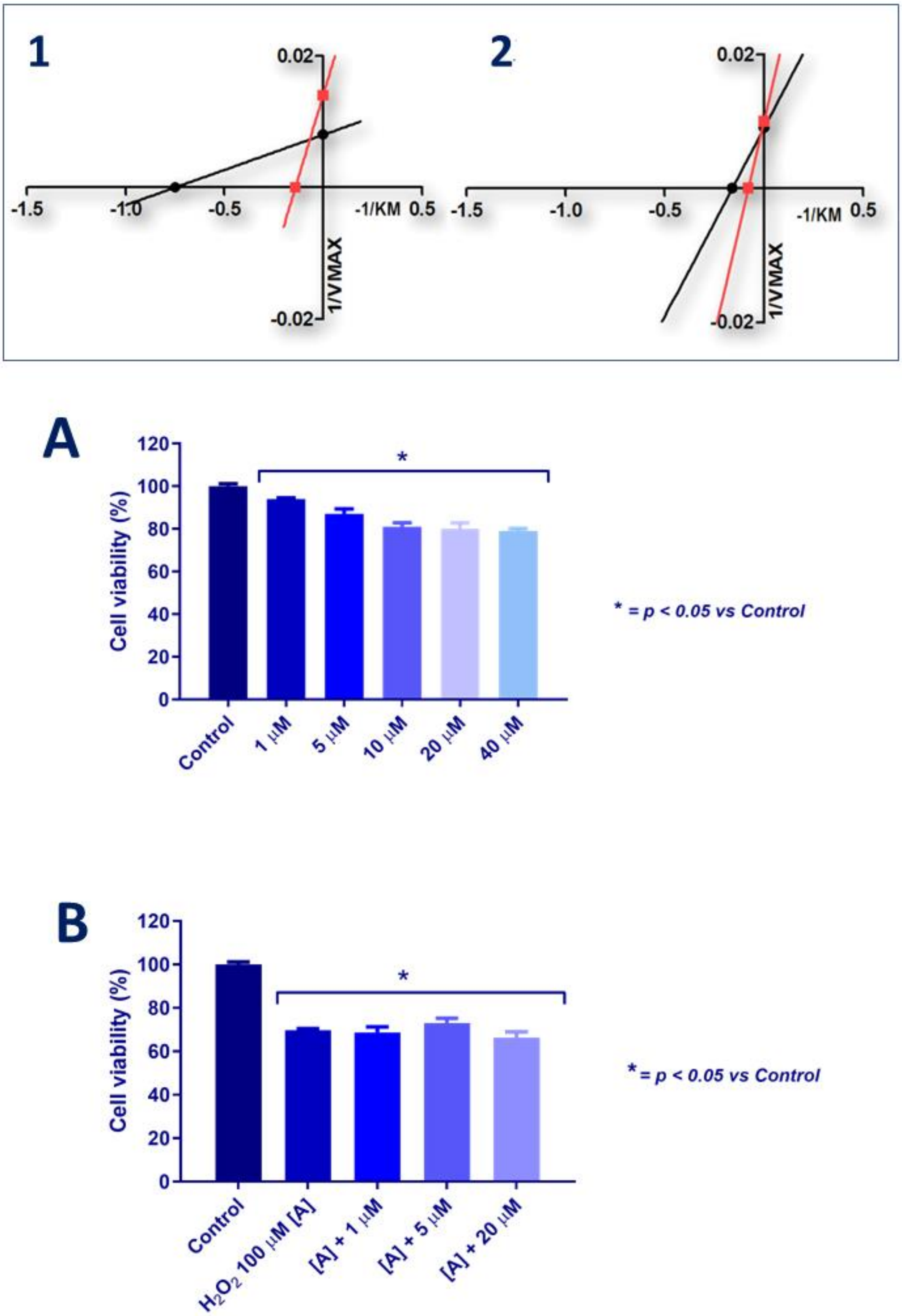

Figure S13: Lineweaver–Burk plot for compound **17** on tyrosinase (inset, 1) and laccase (inset, 2) enzymes. Black line: not inhibited enzyme; red line: inhibited enzyme. Panel A describes the effect of different concentrations of compound **17,** ranging from 1 up to 40 μM, on viability of PC12 cells; Panel B shows the study of potential protective effects of compound **17** on damage induced by H_2O_2 (100 μM). MTT assay was performed on PC12 cells 24 h after each treatment. *$p<0.05$ vs control.

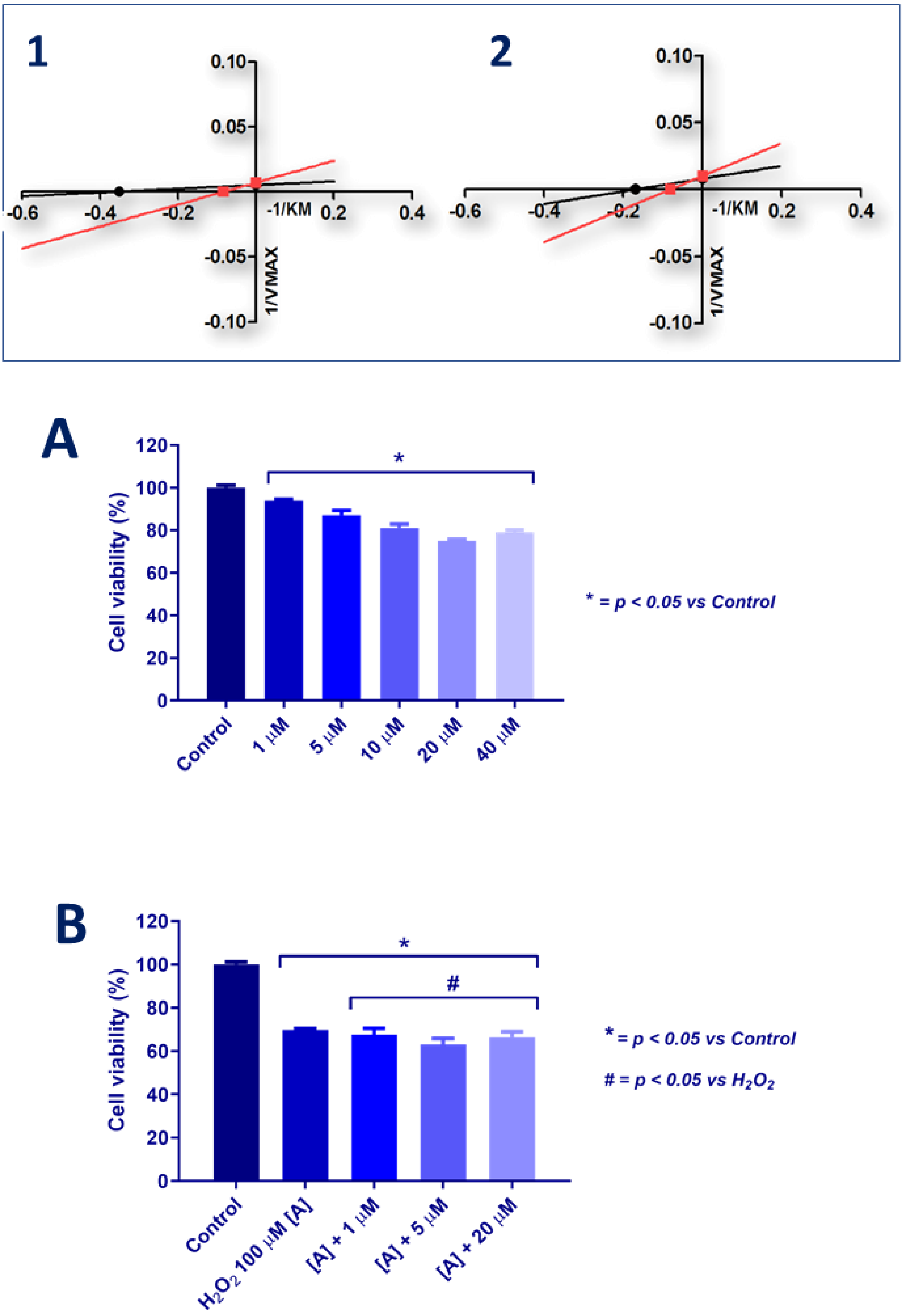

Figure S14: Lineweaver–Burk plot for compound **18** on tyrosinase (inset, 1) and laccase (inset, 2) enzymes. Black line: not inhibited enzyme; red line: inhibited enzyme. Panel A describes the effect of different concentrations of compound **18,** ranging from 1 up to 40 μM, on viability of PC12 cells; Panel B shows the study of potential protective effects of compound **18** on damage induced by H_2O_2 (100 μM). MTT assay was performed on PC12 cells 24 h after each treatment. *p<0.05 vs control.

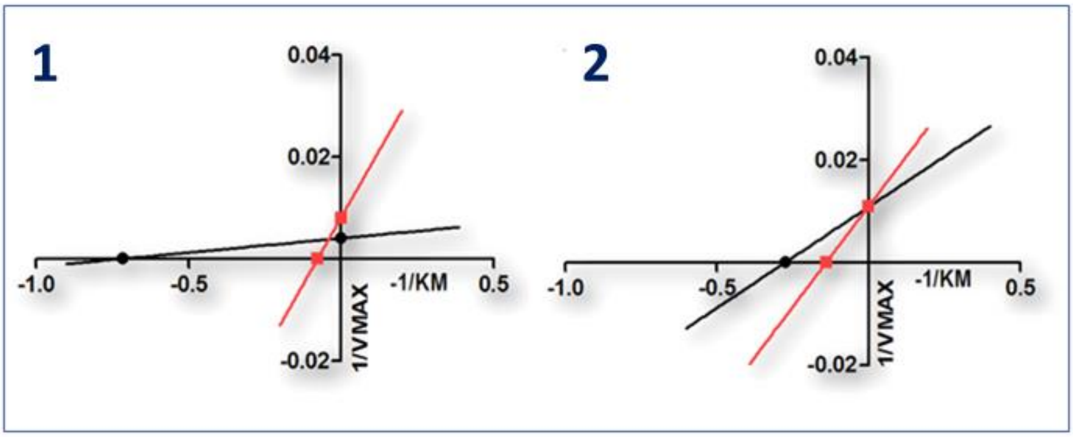

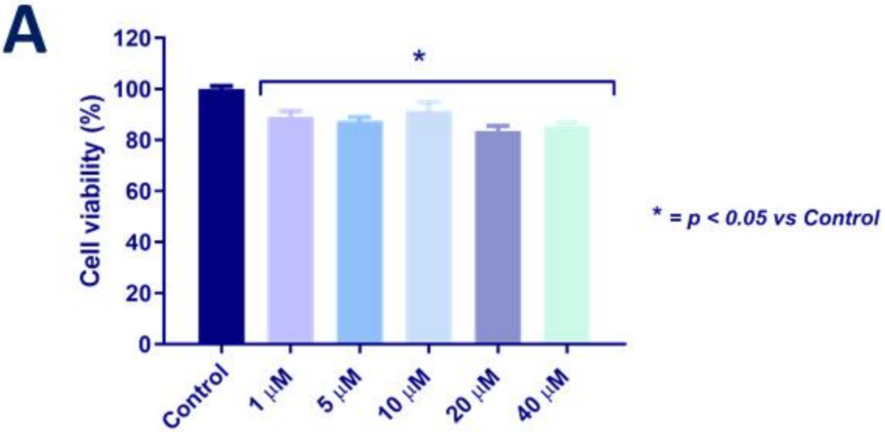

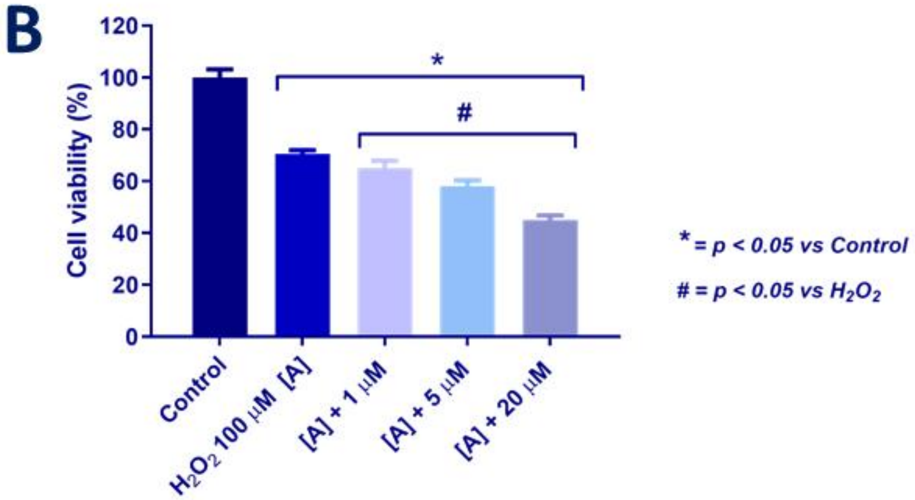

Figure S15: Lineweaver–Burk plot for compound **19** on tyrosinase (inset, 1) and laccase (inset, 2) enzymes. Black line: not inhibited enzyme; red line: inhibited enzyme. Panel A describes the effect of different concentrations of compound **19,** ranging from 1 up to 40 μM, on viability of PC12 cells; Panel B and C show the study of potential protective effects of compound **19** on damage induced by H_2O_2 (100 μM). MTT assay was performed on PC12 cells 24 h after each treatment. *p<0.05 vs control.

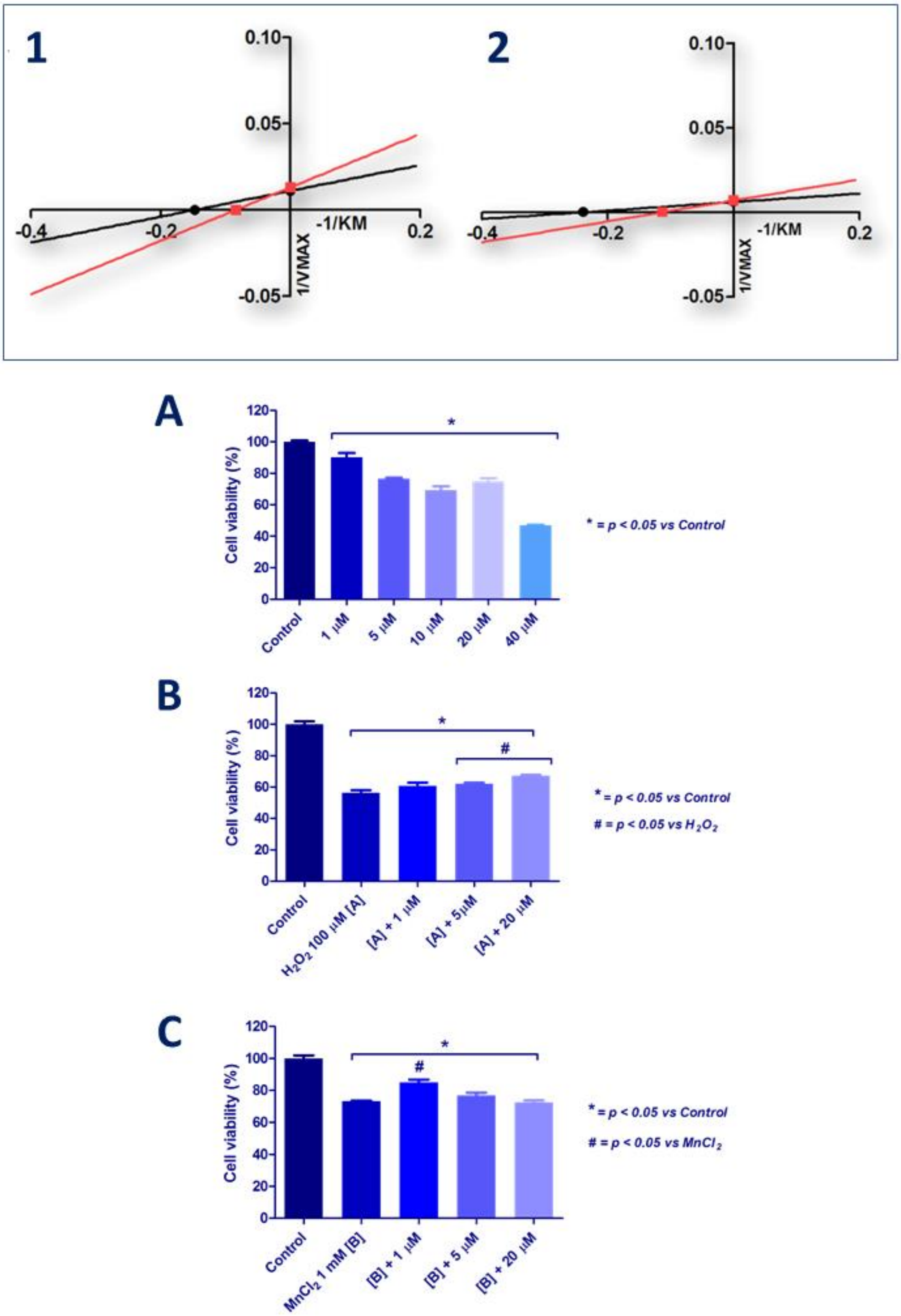

Figure S16: Lineweaver–Burk plot for compound **20** on tyrosinase (inset, 1) and laccase (inset, 2) enzymes. Black line: not inhibited enzyme; red line: inhibited enzyme. Panel A describes the effect of different concentrations of compound **20,** ranging from 1 up to 40 μM, on viability of PC12 cells; Panel B and C show the study of potential protective effects of compound **20** on damage induced by H_2O_2 (100 μM) or $MnCl_2$ (1 mM). MTT assay was performed on PC12 cells 24 h after each treatment. *$p<0.05$ vs control; #$p<0.05$ vs H_2O_2 or $MnCl_2$

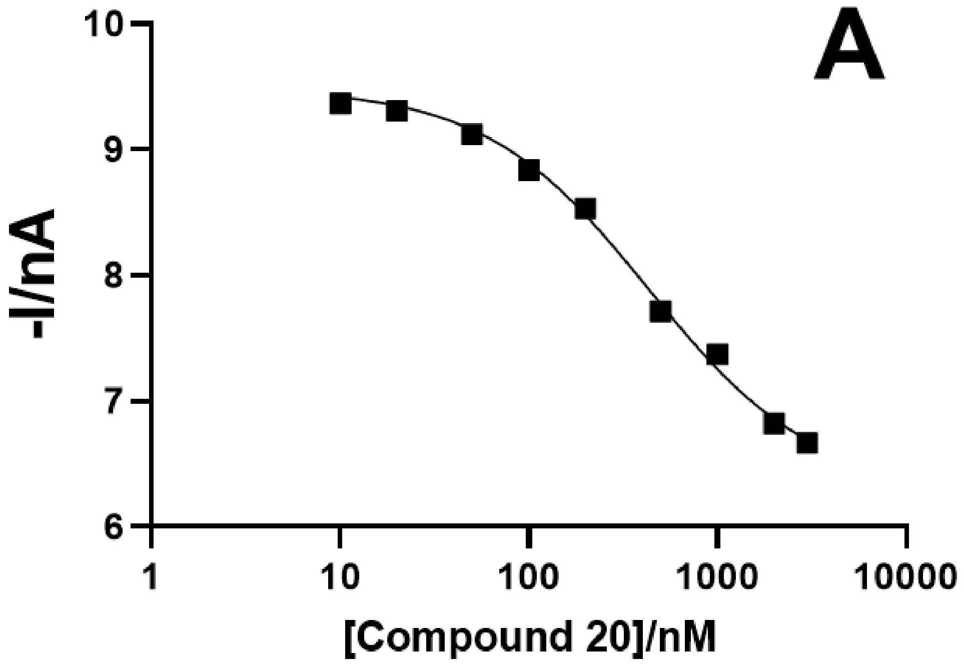

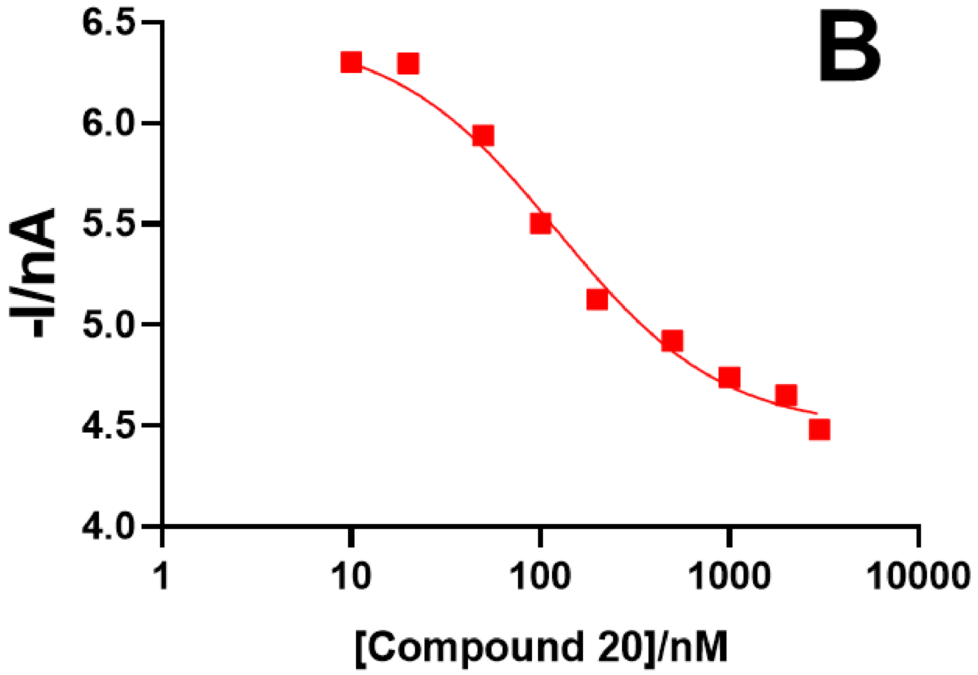

Figure S17: Representative plot of IC_{50} calculation for compound **20**. In panel A is reported the inhibition effect of the studied molecule on tyrosinase enzyme (IC_{50} = 422.7 nM; R^2= 0.996), while in panel B is shown the inhibition on laccase enzyme (IC_{50} = 117.8 nM; R^2= 0.991). Both enzymes were immobilized on the biosensor surface.

1.6 Computational studies

Table S2. Estimated interactions of compounds ***DFB***, ***1*** *and* ***20*** *with the residue of amino acids of Cu1 T1 site of laccase*

Ligand	% Cluster [a]	focc [b]	M.B.E.[c]	E.F.E.B.[d]	E.I.C.,*Ki*[e]	Interaction with amino acids, **H-Bonds**
**DFB**	69	3/3	-5.68	-5,79	56.87 μM	PHE162 PRO163 ASP206 ASN264 PHE265 GLY392 **ALA393** PRO394 ILE455 HIS458
**1**	100	1/1	-5.38	-5,41	108.39 μM	PHE162 **PRO163** ASP206 ASN264 PHE265 GLY392 **ALA393** PRO394 ILE455 HIS458
**20**	25	23/14	-5.58	-6,18	29.54 μM	**PHE162** PRO163 LEU164 ASP206 **ASN264(2)** PHE265 GLY266 GLY392 ALA393 ILE455 HIS458

[a] % Cluster: percentage number of similar conformation grouped by rmsd-tolerance of 2.0 angstrom,
[b] focc: n° of distinct conformational clusters/n° of multi-member conformational clusters, out of 100 runs, using an rmsd-tolerance of 2.0 angstrom,
[c] M.B.E.: Mean Binding Energy,
[d] E.F.E.B.: Estimated Free Energy of Binding,
[e] E.I.C.,Ki: Estimated Inhibition Constant, K*i*,

Table S3. Estimated H-bonds of compounds ***DFB***, ***1*** *and* ***20*** *with the residue of amino acids of Cu1 T1 site of laccase*

Ligands	H-bond	Ligands Atom	Protein Atom	Distance (Å)	Ang. °
**DFB**	1	H7(HD) [a]	ALA393:O(OA) [b]	2,237	132,97
**1**	2	H16(HD)	PRO163:O(OA)	2,126	153,42
		H14(HD)	ALA393:O(OA)	1,803	129,86
**20**	3	H40(HD)	PHE162:O(OA)	1,757	146,24
		H24(HD)	ASN264:OD1(OA)	2,021	159,14
		H26(HD)	ASN264:OD1(OA)	2,153	155,25

[a] (HD) hydrogen donors, [b] (OA) oxygen acceptors

DFB **1** **20**

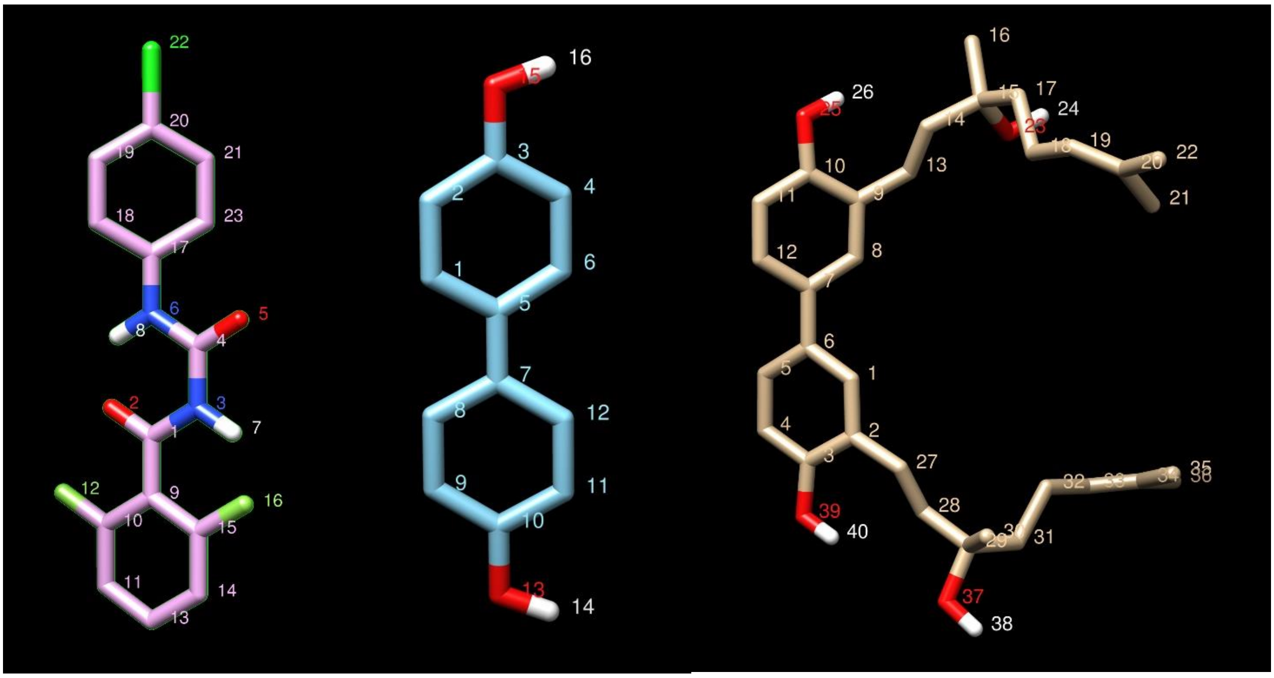

1.7 References

1. Marchiani, A.; Mammi, S.; Siligardi, G.; Hussain, R.; Tessari, I.; Bubacco, L.; Delogu, G.; Fabbri, D.; Dettori, M. A.; Sanna, D.; Dedola, S.; Serra, P.A.; Ruzza, P. Small molecules interacting with a-synuclein: antiaggregating and cytoprotective properties. *Amino Acids* **2013**, *45*, 327-338.

2. Baranovsky, A.; Schmitt, B.; Fowler, J.D.; Schneider, B. *Synth. Commun*. 2006, 1019-1045; b) Chuprajob, T.; Chatchawan, C.; Chokchaisiric, R.; Chunglokd, W.; Sornkaewa, N.; Apichart Suksamrarna, A. Synthesis, cytotoxicity against human oral cancer KB cells and structure–activity relationship studies of trienone analogues of curcuminoids. *Bioorg. Med. Chem. Lett.*, **2014**, *24*, 2839-2844.

3. Jae-Chul, J.; Soyong, J.; Yongam, L.; Dongguk, M.; Eunyoung, L.; Heyin, J.; Miyeon, O.; Seikwan, O.; Mankil, J. Efficient synthesis and neuroprotective effect of substituted 1,3-diphenyl-2-propen-1-ones. *J. Med. Chem*. **2008**, *51*, 4054–4058.

4. Pathak, V.; Ahmad, I.; Kahlon, A.K.; Hasanian, M.; Sharma, S.; Srivastava, K.K.; Sarkar, J.; Shankar, K.; Gupta, A. Syntheses of 2-methoxyestradiol and eugenol template based diarylpropenes as non-steroidal anticancer agents. *RSC Advances*, **2014**, *4*, 35171-35185.

5. Kuo, P.C.; Damu , A. C.; Cherng , C. Y.; Jeng , J.F.; Teng, C.M.; Lee, E.J.; Wu, T.S.
Isolation of a Natural Antioxidant, Dehydrozingerone from Zingiber officinale and Synthesis of Its Analogues for Recognition of Effective Antioxidant and Antityrosinase *Agents Arch Pharm Res* **2005**, *28*, 518-528.

6. Ocasio-Malave’, C.; Donate, M.J.; Sanchez, M.M.; Sosa-Rivera, J.M.; Mooney, J.W.; Perales-DeLeon, T.A.; Carballera, N.M.; Martinez-Ferrer, M.; Sanabria-Rios, D.J. Synthesis of novel 4-Boc-piperidone chalcones and evaluation of their cytotoxic activity against highly-metastatic cancer cells. *Biorg. Med. Chem. Lett.* **2020**, *30, 1,* 126760.

7. Delogu, G.; Dettori, M.A.; Patti, A.; Pedotti, S.; Forni, A.; Casalone, G. Stereoselective oxazaborolidine–borane reduction of biphenylalkyl diketones–lignin models: enantiopure dehydrodiapocynol derivatives. *Tetrahedron:Asymmetry*, **2003**, *14*, 2467-2474.

8. Pisano, M.; Pagnan, G.; Dettori, M.A.; Cossu, S.; Caffa, I.; Sassu, I.; Emionite,L.; Fabbri, D.; Cilli, M.; Pastorino, F.; Palmieri, G.; Delogu, G.; Ponzoni, M.; Rozzo, C. Enhanced anti-tumor activity of a new *curcumin*-related compound against melanoma and neuroblastoma cells. *Mol.Cancer*. **2010**, *9*, 137.

9. Russel W.R.; Scobbie, L; Chesson, A. *Bioorg. & Med. Chem.* Structural modification of phenylpropanoid-derived compounds and the effects on their participation in redox processes. *Bioorg. Med. Chem.* **2005**, *13*, 2537–2546.

10. Bragnier, N.; Guillot, R; Sherrmann, M.C. Diastereoselective addition of sugar radicals to camphorsultam glyoxilic oxime ether: a route toward C-glycosylthreonine and allothreonine. *Org. Biomol. Chem*. **2009**, *7,* 3918-3921.

11. Calia, G.; Rocchitta, G.; Migheli, R.; Puggioni, G.; Spissu, Y.; Bazzu, G.; Mazzarello, V.; Lowry, J.P.; O'Neill, R.D.;Desole, M.S.; Serra, P.A. Biotelemetric monitoring of brain neurochemistry in conscious rats using microsensors and biosensors. *Sensors (Basel)*. **2009**, *9*, 2511-2523.

12. Bazzu, G.; Puggioni, G.G.; Dedola, S.; Calia, G.; Rocchitta, G.; Migheli, R.; Desole, M.S.; Lowry, J.P.; O'Neill, R.D.; Serra, P.A. Real-time monitoring of brain tissue oxygen using a miniaturized biotelemetric device implanted in freely moving rats. *Anal Chem.* **2009**, *81*, 2235-2241.

13. Rocchitta, G.; Migheli, R.; Dedola, S.; Calia, G.; Desole, M.S.; Miele, E.; Lowry, J.P.; O'Neill, R.D.; Derra, P.A. Development of a distributed, fully automated, bidirectional telemetry system for amperometric microsensor and biosensor applications. *Sens. Actuators B Chem* **2007**, *126*, 700-709.

14. Barberis, A.; Garbetta, A.; Cardinali, A.; Bazzu, G.; D'Antuono, I.; Rocchitta, G.; Fadda, A.; Linsalata, V.; D'Hallewin, G.; Serra, P.A.; Minervini, F. Real-time monitoring of glucose and phenols intestinal absorption through an integrated Caco-2TC7cells/biosensors telemetric device: Hypoglycemic effect of fruit phytochemicals. *Biosens Bioelectron*. **2017**, *88*, 159-166.

15. Tamara, H.; Benoît, S.; Stojanc, J.; Weikb, M.; Martyd, J.L; Fourniera, D. Kinetic insight into the mechanism of cholinesterasterase inhibition by aflatoxin B1 to develop biosensors. *Biosens. Bioelectron*, **2009**, 24, 2119–2124.

16. Turdean, G.L. Design and development of biosensors for the detection of heavy metal toxicity. *Int. J. Electrochem.* **2011**, Volume 2011, Article ID 343125.

17. Pinna, A.; Ricco, R.;Migheli, R.; Rocchitta, G.; Serra, P.A.; Falcaro, P.; Malfatti, L.; Innocenzi, P. A MOF-based carrier for in situ dopamine delivery, *RSC Advances* **2018**, *8*, 25664-25672.
